# Five Multimodal AI Configurations Versus Anaesthesiologists for Pecto-Intercostal Block Classification in an Archived-Image Reader Study of a Prospective CABG Cohort

**DOI:** 10.3390/diagnostics16213555

**Published:** 2026-10-28

**Authors:** Anıl Onur, Mesut Öztürk, Korgün Ökmen, Tuğba Onur, Ümran Karaca, Osman Sıla Aydın, Ayşe Neslihan Balkaya, Filiz Ata, İrem Aksoy, Nurullah Keser, Ahmet Kağan As

**Affiliations:** 1 Department of Anesthesiology and Reanimation, University of Health Sciences Bursa Yuksek Ihtisas Training and Research Hospital, Mimar Sinan Mah., Emniyet Cad., 16310 Yıldırım, Bursa, Türkiye; doktormesut@gmail.com (M.Ö.); korgunokmen@gmail.com (K.Ö.); doktor-t@hotmail.com (T.O.); umransuna@hotmail.com (Ü.K.); osmansila@gmail.com (O.S.A.); aynesbalkaya@gmail.com (A.N.B.); filizatafiliz@hotmail.com (F.A.); iremaksoydr@gmail.com (İ.A.); nrllhkeser@hotmail.com (N.K.); 2 Department of Cardiovascular Surgery, University of Health Sciences Bursa Yuksek Ihtisas Training and Research Hospital, Mimar Sinan Mah., Emniyet Cad., 16310 Yıldırım, Bursa, Türkiye; ahmetkagan_as@hotmail.com

**Keywords:** artificial intelligence, multimodal large language models, reader agreement, ultrasound, pecto-intercostal fascial plane block, coronary artery bypass grafting

## Abstract

**Background/Objectives**: We compared five general-purpose multimodal AI configurations with anaesthesiologists classifying sonographic pecto-intercostal fascial plane block spread. **Methods**: This archived-image reader study used a prospective cohort of 120 ASA III adults undergoing isolated coronary artery bypass grafting at one centre. In June 2026, five free AI configurations and three experts classified identical sets of 8–10 left-sided frames. Direct binary comparisons used each expert’s labels in 115 block recipients; five no-block controls were separate. The senior expert reviewed expert labels before setting the final reference, precluding independent expert–AI accuracy comparison. Reference-conditioned balanced accuracy was defined as mean sensitivity and specificity. **Results**: Direct balanced agreement was 42.3–53.9% for four configurations and 55.4–64.9% for Kimi K2.6. AI asserted spread in 17/25 no-block classifications; all 20 human classifications indicated absence. Final-reference balanced accuracy was 76.9–91.9% for experts and 44.6–66.9% for AI, conditional on the expert-informed reference. Pain analyses were exploratory. **Conclusions**: Direct comparisons establish neither anatomical accuracy nor independent expert superiority. Limitations include one centre, operator and ultrasound system; selected frames; free access; no raw AI outputs, reproducible a priori power calculation or non-recruitment-day eligibility log.

## 1. Introduction

Pain after median sternotomy may persist beyond the immediate postoperative period, and chronic poststernotomy pain remains an important sequela [1,2]. Enhanced-recovery guidance recommends multimodal opioid-sparing perioperative care [3]. Regional options for median sternotomy include pecto-intercostal fascial plane block (PIFB), also termed the superficial parasternal intercostal plane block, which deposits local anaesthetic between the pectoralis major and external intercostal muscles to target the anterior cutaneous branches of the thoracic intercostal nerves [4,5]. Contemporary randomised evidence is mixed: a multicentre catheter-based superficial parasternal trial found lower 72 h opioid exposure without improvement in pain or quality of recovery, whereas a separate three-arm trial found no improvement in quality of recovery, pain or analgesic consumption [6,7].

The clinical effect of a fascial plane block depends partly on whether local anaesthetic reaches and spreads within the intended plane. Ultrasound makes tissue planes, the needle path and injectate spread visible, but image acquisition and interpretation remain operator dependent. Purpose-built assistive systems have identified or highlighted sonoanatomical structures with encouraging performance in selected healthy-volunteer, external-validation and simulation settings, and one randomised simulation study suggested improved sustained block-view acquisition among junior anaesthetists [8,9,10,11,12]. These studies did not establish improved clinical block success, fewer complications or better patient outcomes, and substantial variation among expert annotations cautions against treating a single observer as anatomical truth [11].

General-purpose multimodal large language models (LLMs) and related consumer artificial-intelligence (AI) systems can be queried with image inputs without investigator-led task-specific training or fine-tuning. Radiology and ultrasound studies report model- and context-dependent false-positive burdens and fabricated findings [13,14,15,16,17,18,19]. In one radiology study, accuracy also decreased in a 20-image repeat sample assessed 90 days later [16], motivating repeated-session evaluation. Turan et al. reported high ChatGPT-4 accuracy for anatomical landmarks and block-success classification across 147 images from 111 patients with heterogeneous regional blocks [20], but that single-model, mixed-task, raw-accuracy design does not establish performance across products, within a single-block cohort or with deliberately negative no-procedure images. More broadly, single-query evaluations cannot measure within-system repeatability, and between-system disagreement should not be described as within-system unreliability.

We compared five publicly accessible multimodal AI configurations with three experienced anaesthesiologists using the same archived ultrasound image sets and a senior-expert final reference informed by access to the three experts’ classifications. The principal question was agreement with this reference when classifying successful versus not-successful spread in treated participants. Direct comparisons with each individual expert, three-category classifications, five consented no-block image controls and associations with postoperative pain were secondary or exploratory. The study was not designed to establish equivalence or non-inferiority.

## 2. Materials and Methods

### 2.1. Study Design, Setting and Reporting Framework

This single-centre prospective observational cohort was conducted in the Department of Anesthesiology and Reanimation of University of Health Sciences Bursa Yuksek Ihtisas Training and Research Hospital, Bursa, Türkiye. Recruitment and prospective imaging occurred from 1 September 2025 through 27 May 2026; clinical follow-up and data collection were completed by 1 June 2026. The archived-image reading exercise was conducted in June 2026, after enrolment and clinical follow-up had ended.

STARD-AI was the lead reporting framework for the reader study and STROBE for the prospective cohort [21,22,23]. Relevant considerations from CLAIM 2024, TRIPOD-LLM and GRAITE-USRA were also consulted [24,25,26]. These frameworks were applied during reporting, not used as prospective design standards. Supplementary File S2 provides STARD-AI and STROBE checklists, distinguishing reported information from partially met or unmet requirements.

### 2.2. Ethics and Registration

The Medical Sciences Ethics Committee of University of Health Sciences Bursa Yuksek Ihtisas Training and Research Hospital approved the study on 13 August 2025 under institutional protocol no. 2024-TBEK 2025/08-14. On 6 May 2026, the committee approved an amendment establishing the final enrolment target of 120. Participants 81–120 were enrolled only after this amendment had been approved. All participants gave written informed consent before research procedures.

The ClinicalTrials.gov record was first submitted on 6 May 2026 and publicly posted on 12 May 2026 (NCT07581678), after recruitment had begun; registration was retrospective. The original protocol dated 1 August 2025 and the earlier public registry record prioritised the association between anatomical block scores and postoperative NRS. The signed revised protocol dated 29 April 2026 (version 4) instead specified each of five visual-input LLMs’ three-category balanced accuracy against the senior adjudicator’s final reference as primary, with pain associations and comparisons with individual experts secondary. The investigators confirm that this revised protocol accompanied the LLM-method clarification and enrolment increase approved on 6 May, before the June readings. The present report focuses on a narrower descriptive comparison of successful versus not-successful spread in 115 treated participants, with five no-block controls considered separately; this is not the same outcome definition as the protocol’s three-category primary analysis. The registry update submitted on 9 September and publicly posted on 11 September 2026 describes the conducted PIFB-only study, the revised outcome definitions and corrected dates, but does not establish prospective registration of those definitions or of analyses added during revision. Table S4.1 and the accompanying text in Supplementary File S4 distinguish the original protocol, signed April revision, registry history and current analyses. The supporting protocols and committee decisions are supplied separately for confidential editorial review.

### 2.3. Participants

The realised cohort comprised adults aged 18–80 years with American Society of Anesthesiologists (ASA) physical status III undergoing elective isolated coronary artery bypass grafting (CABG) through median sternotomy. Patients were excluded for emergency surgery, redo sternotomy, a minimally invasive or thoracotomy approach, local-anaesthetic allergy, coagulopathy (international normalised ratio > 1.5 or platelet count < 80 × 10^9^/L), infection at the planned injection site, chronic opioid use for >3 months, cognitive dysfunction or pregnancy. Recruitment was limited to days on which the anaesthesiologist who performed PIFB and acquired the ultrasound images was available; on those days, every eligible isolated-CABG patient was invited consecutively.

On recruitment days, 120 patients were assessed, eligible, consented, enrolled and imaged; no eligible invited patient declined study participation. Potentially eligible patients on other days were not logged, so their number cannot be estimated reliably. The investigators attribute faster recruitment after the sample-size amendment to changes in the anaesthesia team’s work organisation, which increased its availability for study-related block performance and ultrasound recording, rather than to an increase in routine surgical volume. The centre’s usual monthly workload is approximately 100–140 open-heart operations. Monthly study enrolment is reported in Supplementary Table S3.20: 80 participants were enrolled from September 2025 through April 2026 and 40 in May 2026. Of the 120 participants, 115 received PIFB and formed the principal reader-analysis cohort. Five declined PIFB but consented to anatomical imaging and research/AI assessment under the authorised study scope. Their inclusion in the common reader series was decided before any rating was observed; their separate control analysis was post hoc. No needle was inserted and no local anaesthetic was administered in these five participants.

### 2.4. Anaesthesia, PIFB and Postoperative Analgesia

General anaesthesia, bispectral-index monitoring and invasive arterial and central venous monitoring followed the institution’s routine practice. PIFB was a routine component of sternotomy analgesia in the department and was not assigned by the research protocol. Every participant in the treated cohort received bilateral PIFB after complete sternal and skin closure. The same consultant anaesthesiologist performed every block at the fourth intercostal space under sterile conditions, using a single injection point on each side and an in-plane technique. Ultrasound guidance used an M5 portable system (Mindray Bio-Medical Electronics, Shenzhen, China) with a 7L4s linear-array transducer at a nominal frequency of 7.5 MHz. The operator was experienced in fascial plane blocks, did not collect the postoperative pain scores and did not participate in the later reader study. Each side received 20 mL of 0.25% bupivacaine (40 mL and 100 mg in total). The solution was prepared by diluting 0.5% bupivacaine (Buvasin; VEM İlaç Sanayi ve Ticaret A.Ş., İstanbul, Türkiye) 1:1 with 0.9% sodium chloride. Spread within the fascial plane was observed in real time, and ultrasound video was recorded using standardised machine settings.

All patients received intravenous paracetamol (Parokan; Haver Farma İlaç A.Ş., İstanbul, Türkiye) 1 g three times daily as scheduled background analgesia. During the first 48 h after surgery, when NRS exceeded 4, rescue treatment with additional paracetamol, tramadol (Ramadex; Haver Trakya İlaç Sanayi ve Ticaret A.Ş., Tekirdağ, Türkiye) or pethidine (Petisel; Haver Farma İlaç A.Ş., İstanbul, Türkiye) was administered at the attending physician’s discretion. Rescue paracetamol was administered in 1 g increments, tramadol in 100 mg increments and pethidine in 50 mg doses. The dataset contained whether each drug was administered during this 48 h interval but not a reliable administration timestamp relative to every NRS measurement; rescue medication was therefore described by reference group but could not be entered as a time-varying covariate in the longitudinal model.

### 2.5. Image Acquisition, Image-Set Construction and De-Identification

Although PIFB was bilateral, only the left side was recorded for the reader study. One researcher, distinct from the block operator and the four human readers, extracted 8–10 representative frames per participant and later conducted the AI queries and recorded the first-response integers. This researcher had no access to postoperative pain data or to the expert readers’ or final-reference classifications. For treated participants, frames covered the pre-block anatomical view, needle advancement, local-anaesthetic spread and final post-injection appearance. Selection was judgement based: frames were not necessarily consecutive, and within each case they were presented in chronological procedural order, but there was no objective temporal-sampling rule or fixed number per stage. Case-level frame counts and per-stage annotations were not retained in the analysis dataset; no retrospective stage annotation or frame-count/needle-frame regression was performed. No-block sets came from left-sided anatomical recordings and lacked needle-advancement and injection frames. All sets were completed before any human or AI classification and were not subsequently altered.

Only Portable Network Graphics (PNG) exports were uploaded. Filenames and image content contained no patient name, protocol number, date of birth, procedure date, hospital number or other direct identifier, and the source video files and any Digital Imaging and Communications in Medicine (DICOM) metadata or headers were not uploaded. No investigator-side cropping, resizing, brightness/contrast adjustment or analytical image normalisation was applied after frame export. The same de-identified PNG frames were supplied to all nine raters.

All raters viewed the 120 participant image sets in the same fixed, non-randomised case order. Within each case, the same 8–10 source PNG frames, in the same chronological procedural order, were provided to every rater. No participant-level age, sex, surgical details, pain scores or other clinical information accompanied the images.

### 2.6. Human Readers and Final Reference Classification

Three anaesthesiologists with 8, 5 and 6 years of fascial-plane-block experience (Experts 1–3) independently assigned one category to each complete image set. None had participated in the anaesthetic or postoperative care of these participants. A fourth anaesthesiologist with 15 years of experience and responsibility for regional-anaesthesia teaching reviewed the same image sets and the three experts’ classifications before stating the final reference category. The human reader assessment took place in June 2026. Analyses used the senior expert’s final categories recorded in the study workbook. This final reference was therefore not independent of the three experts’ classifications. It remained a pragmatic subjective assessment: dermatomal testing was confounded by general anaesthesia and surgery, and no independent anatomical criterion was available for all participants.

Experts 1–3 were blinded to one another’s classifications, AI outputs and clinical outcomes. The senior expert reviewed their classifications before giving the final reference decision and remained blinded to AI outputs and clinical outcomes. All human readers were informed that no-block image sets could be present but were not told their number or positions in the fixed case order. They received the three written category definitions below but no example images, calibration set, training session or consensus meeting. The conducted human-reader forms and AI prompt used the following numerical direction:

1. **Successful:** local anaesthetic distributed completely and clearly within the target fascial plane, with evident hydrodissection.
2. **Patchy/inadequate:** local anaesthetic partly distributed within the plane, with leakage into adjacent tissue and/or poor spread.
3. **Failed/no local anaesthetic:** little or no local anaesthetic visible in the target plane.

All recorded human and AI outputs were analysed under the named categories used in the reader exercise (1, successful; 2, patchy/inadequate; 3, failed/no local anaesthetic). No quantitative or anatomical cut-off was specified for these qualitative categories.

All estimates using the senior-expert final reference are conditional on that expert-informed reference. The additional direct AI–expert comparisons instead use each individual expert’s own labels. Because the senior expert had reviewed Experts 1–3’s labels, comparison of those experts with the final reference is descriptive and cannot independently establish their diagnostic accuracy or superiority over AI. A post hoc comparison substituted the category selected by at least two of Experts 1–3; no three-way split occurred. This majority comparison is not independent validation because it uses the same expert classifications that were available when the final reference was determined.

### 2.7. AI Configurations, Prompt and Query Workflow

Five publicly accessible general-purpose multimodal AI configurations were selected pragmatically for image-input capability and perceived common use. Their reporting labels, based on the investigators’ June 2026 records, were Gemini 3.5 Flash, Claude Sonnet 4.6, GPT-5.5 in ChatGPT, Grok 4.3 and Kimi K2.6. The number and identities were fixed before any output was observed. All 600 queries were completed over several days in June 2026 after clinical follow-up. Exact per-configuration access-date ranges, interface screenshots and provider build identifiers were not retained. These labels therefore describe the configurations selected by the investigators, not independently verified backend versions for every query. Supplementary File S1 distinguishes the recorded labels from unavailable interface evidence.

According to the investigators, each service was used through a free, non-subscription consumer account in its standard web interface under the provider’s default response mode. No investigator-defined system or custom instruction was added. The investigators did not select a non-default response mode. Provider data-use or model-training settings were not actively disabled. Deletion from visible conversation history did not establish deletion from provider back-end systems. Only the de-identified PNG files and the same fixed prompt were supplied. Supplementary File S1 reports the configurations, access route, input format, prompt and response-handling rules.

The same researcher who selected the frames conducted all 600 AI queries and recorded the first-response integers without access to postoperative pain data or to the expert readers’ or reference reader’s classifications. For each configuration and case, this researcher opened a new conversation, uploaded the 8–10 PNG frames together, submitted the unchanged prompt, recorded the first response’s integer, deleted the conversation from visible account history and opened a new conversation for the next case. No response was regenerated or re-prompted, and no study output was used to develop or select the prompt. No formal prompt-development or pretesting dataset was documented. According to the numerical record and investigator account, every first response supplied a valid category. Only the integers were retained, without raw text, screenshots or session logs, so response wording and universal first-response compliance cannot now be independently audited. Account memory and personalisation settings were not recorded or actively modified; new conversations do not prove absence of all provider-level contextual effects.

The prompt stated that images with or without a performed block could be included and required exactly one integer corresponding to the same three conceptual categories given to human readers. The verbatim prompt, including its original typographical error, is reproduced in Supplementary File S1. The investigators supplied no examples and performed no study-specific training, fine-tuning, retrieval augmentation or other adaptation of any system. This was therefore a single-query, out-of-the-box zero-shot workflow.

### 2.8. Outcomes

The principal descriptive analysis in this report was binary balanced accuracy against the senior-expert final reference for successful versus not-successful spread in the 115 treated participants. Reference category 1 was positive; categories 2 and 3 were combined only for this binary task. The signed April protocol had specified three-category balanced accuracy for each LLM. Because no treated participant had reference category 3 and the five no-block controls were not failed attempted injections, the present binary measure and separate control analysis do not constitute an unchanged implementation of that protocol endpoint. No prospectively registered, powered superiority, equivalence or non-inferiority comparison is claimed. Three-category classifications and within-group agreement are reported descriptively in the treated cohort. The additional direct comparisons with individual experts, paired AI-expert differences, expert-majority comparison and longitudinal pain models are exploratory; the latter models were added after the protocol, although the general pain-association aim was prespecified as secondary in April. In the no-block controls, categories 2 and 3 remain distinct because category 2 asserts partial local-anaesthetic spread. Descriptive matrices combining all 120 sets are confined to the supplement and cannot establish recognition of genuine procedural failures. For pain, the exposure was the final reference classification of the left-side images, not established bilateral block success. Time to first mobilisation was planned but not collected; the earlier registered 24 h analgesic-consumption outcome cannot be reconstructed from the available 48 h receipt data and unreliable administration timestamps.

Resting and coughing numerical rating scale (NRS) assessments were scheduled at 12, 24 and 48 h after surgery. An assessment at 6 h was also planned, but it could be obtained only in patients who had already been extubated by then and was therefore not included in the analyses. The forms recorded the postoperative assessment time point and NRS score, not separate clock-time timestamps; 12, 24 and 48 h refer to time after surgery, not time since extubation. NRS ranged from 0 (no pain) to 10 (worst imaginable pain), recorded verbally as whole numbers by a clinical team separate from the block operator and later readers and without access to image classifications. Preoperative anxiety and depression were assessed with HADS [27]. Ventilation prevented some pain assessments. Observed scores were used in the main exploratory models, with missing-data sensitivity analyses described below. Rescue receipt covered the first 48 h. Complications and mortality were summarised from the index-admission record; standardised dates and severity grades for non-fatal events and a separate PIFB-specific surveillance field were unavailable.

### 2.9. Study Size

The reader study used all image sets from the final approved 120-participant cohort. The original protocol cited a pilot-based calculation, and the signed April revision asserted that increasing enrolment from 80 to 120 would provide power above 90%, but the assumptions needed to reconstruct these calculations were not supplied. Neither statement provides a reproducible a priori justification for the present treated-cohort binary reader comparison. That comparison contained 86 reference-positive and only 29 reference-negative cases. Achieved specificity interval widths ranged from 16.6 to 34.0 percentage points across readers; for Expert 2, specificity was 65.5% (95% CI 47.3–80.1), an unrounded width of 32.7 points. Balanced-accuracy interval widths ranged from 8.7 to 20.3 points. These are precision descriptions of the observed sample, not post hoc power or evidence that small differences can be excluded.

### 2.10. Statistical Analysis

Continuous variables are summarised as mean ± standard deviation or median (interquartile range), and categorical variables as *n* (%). Table 1 gives variable-specific unavailable counts and identifies presence-coded fields. The principal reader metric was balanced accuracy; sensitivity, specificity, accuracy and agreement summaries were secondary. PPV and NPV are presented in Supplementary File S3 because they depend on the reference-defined category prevalence.

For the binary task, sensitivity and specificity refer to agreement with reference-positive and reference-negative labels, respectively; accuracy is the overall binary match proportion and balanced accuracy is (sensitivity + specificity)/2. These quantities do not establish anatomical diagnostic accuracy. Wilson 95% intervals were used for individual proportions and 10,000 reference-stratified participant bootstrap resamples for balanced accuracy and paired AI-minus-expert differences. The same sampled participants were used for both readers in each paired contrast. The 15 paired contrasts are reported descriptively as differences and pointwise confidence intervals, without *p* values. Confidence intervals remain pointwise, not simultaneous. These comparisons condition on the recorded final reference; resampling does not remove the dependence created by access to the experts’ labels during reference determination. No probability outputs were available for empirical ROC or calibration analysis.

As an additional exploratory comparison, each AI configuration was assessed against each of Experts 1–3’s own labels in the same 115 treated participants, without using the senior-expert final reference. Expert category 1 was the positive comparison label; categories 2 and 3 were combined as not successful. The same balanced-accuracy calculation was used, here interpreted as class-balanced agreement with a subjective expert comparator rather than anatomical accuracy. Pointwise 95% percentile intervals used 10,000 whole-participant bootstrap resamples stratified by the selected expert’s binary category; all classifications from a sampled participant were retained together. These estimates are conditional on that expert’s recorded labels and empirical class distribution. They are not multiplicity-adjusted superiority tests; no additional hypothesis tests were performed. Underlying counts, seeds and sparse-cell limitations are in Supplementary Table S3.19 and the accompanying text.

Three-category exact agreement and linearly weighted Cohen κ were calculated in the 115 treated cases; 95% κ intervals used 10,000 whole-participant bootstrap resamples. All-120 confusion matrices and coefficients are supplementary descriptions of a constructed mixture that includes five structurally different no-block controls. Fleiss κ, complete agreement and mean pairwise observed agreement describe the AI and expert groups separately; unequal group sizes preclude interpreting their difference as a matched test. A negative Fleiss κ can reflect heterogeneous marginal response tendencies under its pooled-marginal chance model rather than universal disagreement. A fixed-margin permutation benchmark is summarised descriptively without an additional significance claim. Sex- and BMI-stratified error counts are exploratory descriptions, not powered fairness comparisons.

Exploratory pain analyses used separate cumulative-logit mixed models for resting and coughing NRS, with participant random intercepts, categorical time, reference-defined spread group, their interaction, sex, age, BMI, intraoperative fentanyl, cross-clamp time and HADS anxiety and depression. For the ordinal models, association direction and exploratory pointwise inference are reported rather than conditional odds-ratio magnitudes; these findings do not quantify NRS-point differences or clinical benefit. These post hoc models assume proportional odds and conditional missing at random (MAR). Gaussian mixed-model and expanded-confounder estimates were supplementary comparisons. A delta-adjusted ordinal sensitivity analysis considered hypothetical unobserved responses at eight temporarily unavailable visits among the 113 participants with at least one NRS. Selected log-odds shifts corresponded to 0.25-, 0.5-, 1-, 2- or 4-fold odds of higher pain; 50 imputed datasets per scenario were pooled on the log-odds scale. The two never-extubated participants were excluded from both modelling and imputation. This sensitivity analysis does not reconstruct pain during ventilation. Full assumptions, numerical checks and method sources are in Supplementary File S3. Rescue timing was unavailable for time-varying adjustment. Individual-rater mean-NRS summaries were retained descriptively without the redundant correlation tests.

All nine readers had a recorded category for every image set. Supplementary File S3 gives analysis populations, coding, missingness, resampling settings and exploratory sensitivity analyses. Earlier descriptive analyses used IBM SPSS Statistics 26 (IBM Corp., Armonk, NY, USA); reproducible reader and regression analyses used Python 3.12.14 with NumPy, pandas, SciPy and statsmodels. Package versions, seeds and numerical convergence checks are retained with the analysis code. Analyses added after the protocol are identified as post hoc.

### 2.11. Use of Generative AI in Manuscript Preparation

OpenAI Codex assisted with language editing, manuscript restructuring, analysis-code drafting, table preparation and cross-document consistency checks during preparation and revision. It was not used to generate patient observations, ultrasound images or the recorded June reader classifications. The authors reviewed the code, outputs and scientific text and take responsibility for the submitted content. This manuscript-preparation assistance is distinct from the five AI configurations evaluated as study readers.

## 3. Results

### 3.1. Participant Flow and Baseline Characteristics

All 120 enrolled participants supplied complete image sets and nine recorded classifications, yielding 1080 classifications (Figure 1). The 115 treated participants contributed 1035 classifications; the five no-block controls contributed 45. Pain observations were available for 107, 111 and 113 treated participants at nominal 12, 24 and 48 h. Six participants had not yet been extubated at 12 h, including two who remained ventilated at 24 h. Two other participants were never extubated, had no NRS observations and were the two patients who died on ICU days 10 and 13. Each longitudinal pain outcome therefore had 331 observations from 113 participants.

The 115 treated participants had a mean age of 60.0 ± 9.1 years; 89 (77.4%) were male and all had ASA physical status III. Baseline and perioperative features are shown in Table 1; corresponding characteristics by senior-expert final reference-defined left-side spread group, with descriptive standardised differences and no significance testing, are reported in Supplementary Table S3.17B. At least one postoperative complication was recorded for 25/115 (21.7%) participants: delirium in 12, need for non-invasive ventilation in six (including two with atelectasis), surgical revision in four, pneumonia in one, arrhythmia with sepsis in one and embolectomy in one. Two participants died after cardiac-surgical complications on intensive-care days 10 and 13, one in each reference-defined spread group. Median intensive-care stay was 2 days (interquartile range [IQR] 1–2) and hospital stay was 5 days (IQR 4–6).

### 3.2. Reference Classifications and Rater Distributions

Within the 115 treated participants, the senior-expert final reference classified 86 (74.8%) image sets as successful and 29 (25.2%) as patchy/inadequate; none was classified as failed/no local anaesthetic. The five no-block image sets were all classified as failed/no local anaesthetic by the senior-expert final reference and all three non-reference experts.

Table 2 reports the treated-cohort three-category distributions and reference agreement. Linear-weighted κ ranged from −0.094 to 0.210 for AI configurations and from 0.539 to 0.820 for experts. No treated participant had reference category 3, so category-3 sensitivity for an actual failed injection cannot be estimated. All-120 matrices, including the five controls, are retained in Supplementary File S3 solely as descriptive counts.

Among the 115 treated participants, three-category Fleiss κ was 0.324 (95% CI, 0.193–0.451) for the three experts and −0.101 (95% CI, −0.133 to −0.067) for the five AI configurations. Complete agreement occurred in 70/115 and 1/115 cases, respectively. These coefficients describe agreement across readers or configurations, not repeatability within an AI configuration. Binary and combined all-120 descriptive results and their marginal-frequency interpretation are in Supplementary File S3.

### 3.3. Expert Vote Patterns

The three-expert majority equalled the senior-expert final reference in all 115 treated cases. All three experts agreed in 70 cases; in each of the other 45, the final reference matched the two-expert majority and exactly one expert differed. Experts 1, 2 and 3 accounted for 8, 20 and 17 discordances, respectively, without overlap between their discordant case sets. No treated case had two experts departing from the final reference. Substitution of the identical majority labels necessarily left the AI estimates unchanged. The all-120 vote-pattern table is supplied in Supplementary File S3. The senior expert’s access to Experts 1–3’s labels before the final decision means that agreement with this reference is not independent validation; the recorded final classifications alone do not establish how each decision was reached.

### 3.4. Binary Final-Reference Estimates and Direct AI–Expert Agreement

Balanced accuracy against the reference was 76.9–91.9% for experts and 44.6–66.9% for AI configurations (Table 3; Figure 2A). Four AI intervals included 50%; this is not evidence of equivalence to chance. Operating tendencies differed: Grok assigned category 1 to 108/115 treated cases, whereas GPT-5.5 did so in 14/115.

All 15 paired balanced-accuracy differences showed closer expert agreement with the final reference, ranging from −47.3 to −10.1 percentage points (Figure 2B). The Kimi K2.6 versus Expert 2 contrast was −10.1 points (95% CI −21.6 to 1.5). Supplementary File S3 includes all paired estimates and descriptive sex/BMI error summaries.

In direct comparisons with Experts 1–3 separately, balanced agreement ranged from 42.3 to 53.9% for Gemini, Claude, GPT and Grok and from 55.4 to 64.9% for Kimi K2.6 (Table 4). Each column uses one expert’s labels, not the senior-expert final reference, and its intervals are conditional descriptive summaries. Raw agreement need not track balanced agreement: Grok matched Expert 1 in 81/115 binary classifications (70.4%) despite a balanced agreement of 50.2%, because of its tendency to select the successful category. Supplementary Table S3.19 supplies all underlying binary counts. These comparisons do not establish anatomical accuracy, equivalence or an inferential ranking of AI configurations.

Among the three experts, exploratory binary agreement ranged from 67.8% to 78.3%, and directional balanced agreement ranged from 57.7% to 72.4% (Supplementary Table S3.19B).

### 3.5. Exploratory No-Block Image Controls

No needle was inserted and no local anaesthetic was administered in the five no-block controls. The senior-expert final reference and three experts returned category 3 for all five image sets (20/20 human classifications). Across the five AI configurations, only 8/25 (32.0%) classifications selected category 3. Nine (36.0%) selected category 2, thereby asserting partial local-anaesthetic spread, and eight (32.0%) selected category 1, thereby asserting a successful block (Table 5). Thus, 17/25 (68.0%) AI responses asserted that local anaesthetic was present when none had been injected.

Correct category-3 classifications were returned by GPT-5.5 for 4/5 controls, Gemini 3.5 Flash and Kimi K2.6 for 2/5 each, and Claude Sonnet 4.6 and Grok 4.3 for 0/5 each. Successful-block assertions were returned for 1/5 controls by Gemini, 2/5 by Claude and 5/5 by Grok; GPT-5.5 and Kimi K2.6 returned none. These five participants were not attempted-block failures, and the small post hoc set cannot estimate a general false-positive or safety-event rate. It demonstrates only that the tested image-only configurations sometimes asserted injectate spread in deliberately no-block images despite the prompt warning that such cases might be present.

### 3.6. Postoperative Pain and Rescue Analgesia

Postoperative pain findings are detailed in Supplementary File S3. Both ordinal and Gaussian models associated reference-successful spread with lower pain at 24–48 h, but the magnitude depends on the model and estimand. The ordinal results are presented as association direction and exploratory pointwise inference, not as estimates of clinical benefit. Later associations persisted under the selected hypothetical missingness shifts; resting pain at 12 h was sensitive to model specification and the fourfold-higher-pain sensitivity scenario. Rescue receipt during the first 48 h in reference-successful versus patchy/inadequate groups was 27/86 versus 10/29 for paracetamol, 16/86 versus 1/29 for tramadol and 2/86 versus 2/29 for pethidine. More tramadol use in the successful group and unavailable administration timing limit attribution of later pain differences to block spread. The derived median interval of 6.2 h was calculated as nominal 12 h minus time to extubation, not from actual pain-assessment timestamps.

## 4. Discussion

In this archived-image reader study, the three anaesthesiologists agreed more closely than the five tested consumer AI configurations with the senior-expert final reference. However, the senior expert reviewed their classifications before setting that reference; expert agreement may therefore be favoured, and the comparison cannot independently establish expert superiority. Paired resampling describes uncertainty in these reference-conditioned differences but does not remove the dependence. The majority matched the final reference in all 115 treated cases, with all 45 discordances confined to non-overlapping single-reader case sets. This pattern neither validates the reference nor establishes how review of the experts’ labels affected individual decisions, and substituting the identical majority labels adds no independent evidence. In a different fascial-plane setting, two blinded readers assessing ultrasound localisation after deep rectus sheath block agreed on 53/60 sites (κ = 0.49), illustrating reader dependence without validating the present rubric [28].

The additional comparisons with individual experts avoid using the expert-informed final reference, while retaining subjective comparison labels. Four AI configurations had direct balanced agreement near 0.5; Kimi had higher point estimates, which varied by expert and had appreciable uncertainty. This complements, rather than independently validates, the final-reference results. It does not support a uniform claim that every AI configuration is diagnostically inferior to every expert. Class-balanced and raw agreement should be distinguished, as illustrated by Grok’s high raw agreement but near-0.5 balanced agreement with Expert 1.

The clinical and technical tasks should remain distinct. PIFB is used for sternotomy analgesia, but randomised trials and pooled evidence report heterogeneous effects on postoperative pain [4,5,6,7,29]. Sonographic spread is not a validated surrogate for the extent or duration of bilateral analgesia. Task-specific ultrasound guidance systems have addressed different targets and workflows [8,9,10,11,12]; their results cannot be transferred to general-purpose consumer configurations. Likewise, performance in musculoskeletal, breast or thyroid ultrasound and other image tasks is setting dependent [14,15,16,17,18,19,30]. The perioperative antithrombotic scenario study evaluated text-based decisions rather than ultrasound spread interpretation [31].

The AI findings are a snapshot of free consumer configurations accessed in June 2026. One query per case cannot measure within-configuration repeatability. Raw responses, exact query dates, interface screenshots and backend identifiers were not retained; universal first-response compliance therefore rests on the investigators’ account and integer record. Formal prompt-development or pretesting data were not documented, and the effects of its “Facial” typographical error and non-standard “USG” abbreviation were not tested. Unrecorded personalisation and changing provider defaults further limit replication. Evidence on task-specific regional-anaesthesia models or general medical knowledge does not remedy these auditability limitations [13,20].

The five no-block sets were consented anatomical image controls, not failed attempted blocks. Their lack of needle-advancement and injection frames made them structurally recognisable, particularly to experienced human readers. Seventeen of 25 AI classifications nevertheless asserted partial or complete spread. This small, post hoc control analysis identifies errors in the tested image sets; it cannot estimate a population hallucination rate, general clinical safety or sensitivity to genuine procedural failure. Subjective frame selection also embedded the selector’s judgement in every reader’s input. The retained integer outputs do not show whether any AI configuration used frame count, needle visibility or another specific visual cue. Without case-level stage annotations or repeat assessment under fixed-interval sampling, the influence of frame selection cannot be quantified.

Postoperative pain was a separate exploratory association, not validation of the image reference. Lower pain associated with reference-defined successful spread became more apparent at 24–48 h rather than immediately, and rescue tramadol was more frequent in that group. Unrecorded rescue timing, later care, confounding, left-side classification of a bilateral procedure and ventilation-related missingness prevent a causal attribution to the block. Ordinal and missing-data sensitivity analyses assess specified modelling choices but cannot recover pain while patients were not assessable. Between-group contrasts also should not be equated with within-patient minimal clinically important change thresholds, which vary across settings [32,33,34].

The study used identical image content and order for all readers, complete recorded classifications and directly paired descriptive comparisons. Its scope remains limited to one centre, one operator, one ultrasound system and ASA III adults undergoing isolated CABG. Recruitment depended on team availability, with no log of eligible patients on other days. The fixed case order, qualitative rubric without calibration, only 29 reference-negative treated cases and absence of an independent anatomical standard limit precision and generalisability. The present treated-cohort binary comparison is narrower than the three-category LLM endpoint in the signed April protocol, and registration was retrospective. Future studies should prospectively define an independent reference, outcome and sampling rules, retain auditable query records and include external cohorts and repeated readings.

Only de-identified PNG images were uploaded, but provider data-use settings were not actively disabled. Participant consent and Ethics Committee scope should not be interpreted as proof of provider-side deletion or a substitute for explicit data-governance arrangements in future clinical deployment.

## 5. Conclusions

In the 115 treated participants, direct comparison with each expert separately showed binary balanced agreement of 42.3–53.9% for four June 2026 consumer AI configurations and 55.4–64.9% for Kimi K2.6. For context, expert–expert directional binary balanced agreement ranged from 57.7% to 72.4% (Supplementary Table S3.19B). In five exploratory no-block image controls, 17/25 AI classifications asserted partial or complete local-anaesthetic spread, whereas all 20 human classifications indicated its absence. These findings concern agreement with subjective reader labels and responses to selected no-block images, not independent anatomical accuracy or expert diagnostic superiority. The final-reference comparisons remain conditional on an expert-informed, non-independent reference. The study does not establish autonomous clinical suitability or performance of other AI configurations.

## Figures and Tables

**Figure 1:**
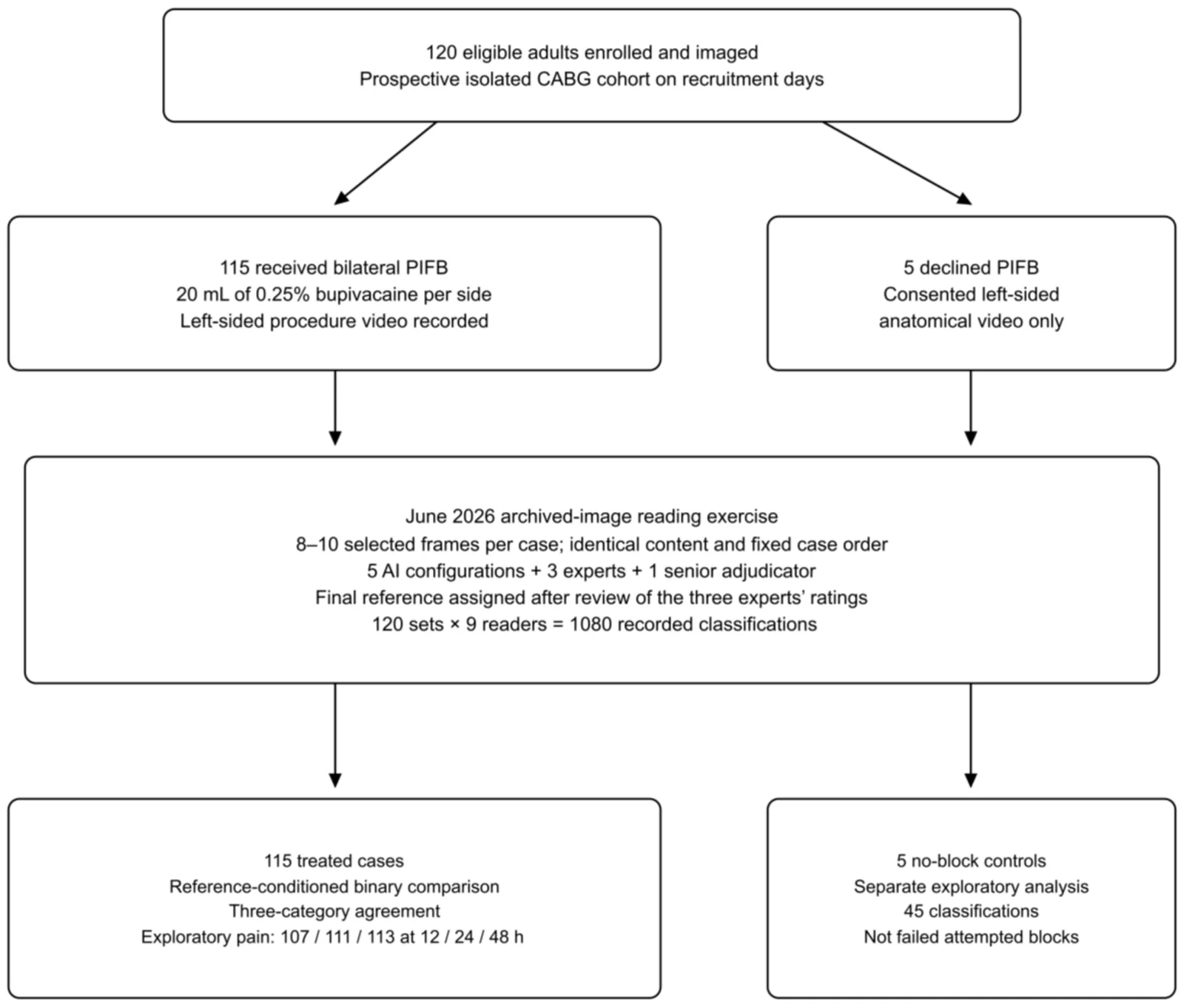
Study flow. Although 115 participants received bilateral pecto-intercostal fascial plane blocks (PIFB), all readers assessed only selected frames from the left-sided recording. No-block controls were analysed separately from the primary treated cohort. AI denotes artificial intelligence.

**Figure 2:**
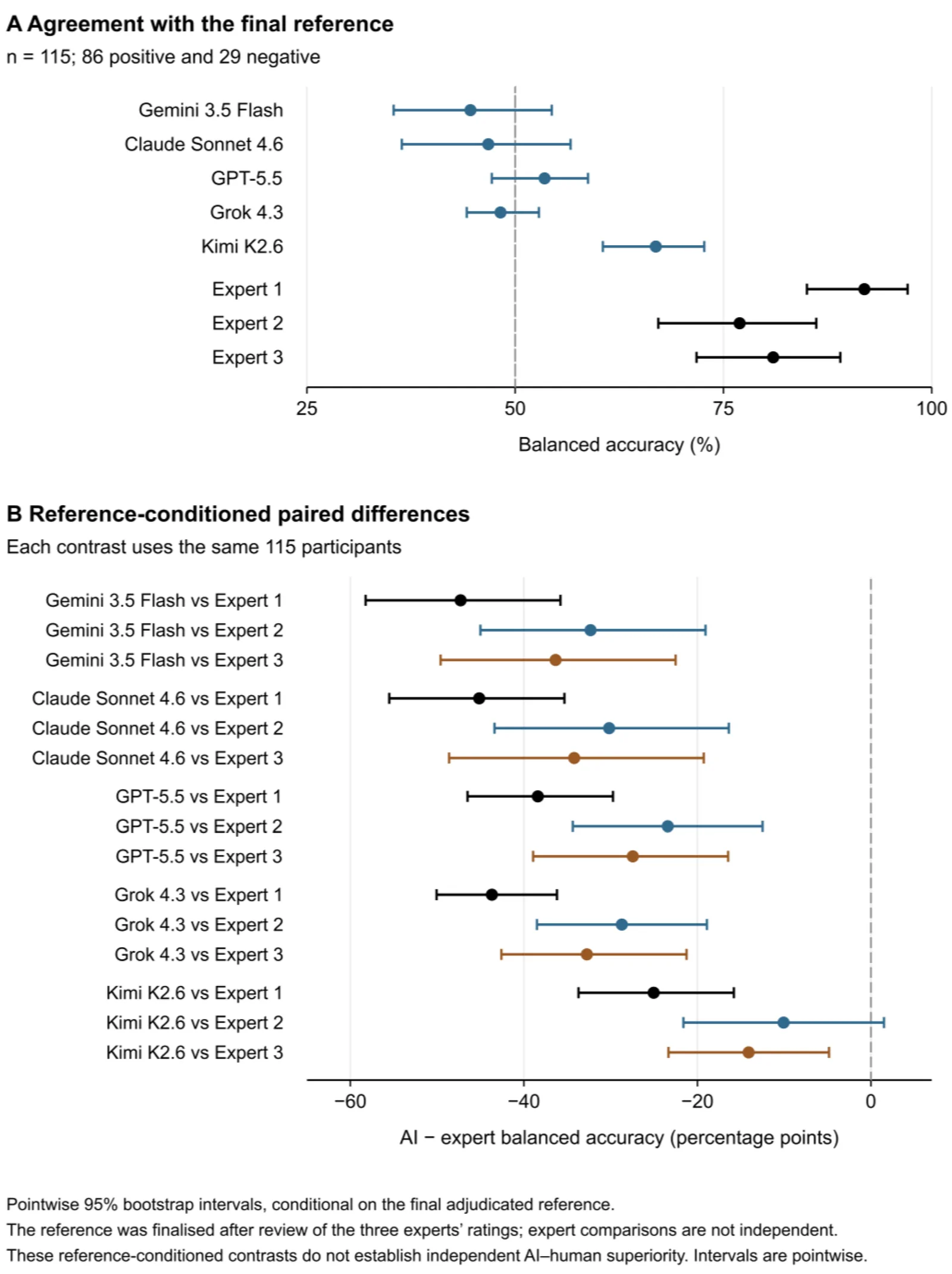
Reader agreement with the senior-expert final reference in 115 treated participants. (**A**) Balanced accuracy, with 95% reference-stratified bootstrap intervals; the 50% line is a descriptive reference. (**B**) Paired AI-minus-expert differences in balanced accuracy, in percentage points, with pointwise 95% bootstrap intervals. Every contrast uses the same 115 participants (86 reference-positive, 29 reference-negative). Intervals are pointwise rather than simultaneous; paired *p* values are not reported. Negative differences indicate closer agreement by the expert reader with the final reference, which was determined after the senior expert had seen the experts’ labels. They do not establish independent diagnostic superiority. AI labels identify the reported June 2026 consumer configurations, not verified backend builds. In panel A, blue denotes AI configurations and black denotes expert readers; in panel B, black, blue and brown denote comparisons with Experts 1, 2 and 3, respectively.

**Table 1:** Baseline demographic and clinical characteristics of the treated cohort (*n* = 115).

Characteristic	Category/Statistic	Value	Unavailable *n*
Age, years	Mean ± SD	60.0 ± 9.1	0
BMI, kg/m^2^	Median (IQR)	27.10 (25.40–28.71)	0
Sex	Male	89 (77.4%)	0
	Female	26 (22.6%)	0
Education	Primary school	10 (8.7%)	0
	Secondary school	20 (17.4%)	0
	High school	75 (65.2%)	0
	University	10 (8.7%)	0
ASA physical status III		115 (100%)	0
Hypertension		78 (67.8%)	0 *
Diabetes mellitus		57 (49.6%)	0
Asthma		2 (1.7%)	0 *
Alcohol use		3 (2.6%)	0 *
Smoking		54 (47.0%)	0
Ejection fraction, %	Median (IQR)	55 (50–60)	0
EuroSCORE II	Median (IQR)	1.0 (1.0–1.0)	0
HADS anxiety	Median (IQR)	8 (5–9)	0
HADS depression	Median (IQR)	5 (3–8)	0
Cross-clamp time, min	Mean ± SD	56.2 ± 17.6	0
Intraoperative fentanyl, μg	Mean ± SD	533.0 ± 120.6	0
Time to extubation, min (*n* = 113)	Median (IQR)	360 (290–420)	2
Intensive-care stay, days	Median (IQR)	2 (1–2)	0
Hospital stay, days	Median (IQR)	5 (4–6)	0
Any postoperative complication		25 (21.7%)	0 *
Mortality		2 (1.7%)	0 *

Unless otherwise stated, the denominator was 115. Time to extubation was available for 113 participants; two participants were not extubated. Race and ethnicity were not collected. Abbreviations: ASA, American Society of Anesthesiologists; BMI, body mass index; EuroSCORE II, European System for Cardiac Operative Risk Evaluation II; HADS, Hospital Anxiety and Depression Scale; IQR, interquartile range; SD, standard deviation. Unavailable counts refer to the analysed variables. EuroSCORE II values in the available source column were whole-number valued; one-decimal presentation does not recover additional measurement precision. * In the remaining presence-coded fields, source blanks were interpreted as absence under the original coding convention, not individually verified negative observations. Diabetes and smoking use the investigators’ confirmed present/absent coding. Original source-blank counts are supplied in Supplementary Table S3.10. Weight and height each had 45 source blanks although recorded BMI was complete. The two unavailable extubation durations were in the never-extubated participants.

**Table 2:** Three-category classification and agreement with the senior-expert final reference in the 115 treated participants.

Reader	Category 1 *n*	Category 2 *n*	Category 3 *n*	Exact Agreement % (95% CI)	Weighted κ (95% CI)
Gemini 3.5 Flash	78	35	2	54.8 (45.7–63.6)	−0.094 (−0.249–0.075)
Claude Sonnet 4.6	46	69	0	42.6 (34.0–51.7)	−0.044 (−0.189–0.103)
GPT-5.5	14	87	14	31.3 (23.5–40.3)	0.033 (−0.025–0.090)
Grok 4.3	108	7	0	70.4 (61.5–78.0)	−0.047 (−0.142–0.077)
Kimi K2.6	33	82	0	52.2 (43.1–61.1)	0.210 (0.117–0.315)
Expert 1	84	31	0	93.0 (86.9–96.4)	0.820 (0.686–0.930)
Expert 2	86	29	0	82.6 (74.7–88.5)	0.539 (0.342–0.705)
Expert 3	85	30	0	85.2 (77.6–90.6)	0.612 (0.437–0.772)

Values refer only to treated participants. Categories: 1, successful spread; 2, patchy/inadequate spread; 3, failed/no local anaesthetic (LA). The reference assigned 86 cases to category 1, 29 to category 2 and none to category 3. Exact agreement uses Wilson 95% intervals; linear-weighted Cohen κ uses 10,000 participant bootstrap resamples. These are reference-conditioned agreement measures, not anatomical diagnostic accuracy. The senior expert reviewed Experts 1–3’s classifications before finalising the reference; their agreement with it is not an independent accuracy estimate.

**Table 3:** Binary classification agreement with the senior-expert final reference in the 115 treated participants.

Reader	Sensitivity %	Specificity %	Accuracy %	Balanced Accuracy %
Gemini 3.5 Flash	65.1 (54.6–74.3)	24.1 (12.2–42.1)	54.8 (45.7–63.6)	44.6 (35.4–54.4)
Claude Sonnet 4.6	38.4 (28.8–48.9)	55.2 (37.5–71.6)	42.6 (34.0–51.7)	46.8 (36.4–56.6)
GPT-5.5	14.0 (8.2–22.8)	93.1 (78.0–98.1)	33.9 (25.9–43.0)	53.5 (47.2–58.7)
Grok 4.3	93.0 (85.6–96.8)	3.4 (0.6–17.2)	70.4 (61.5–78.0)	48.2 (44.2–52.8)
Kimi K2.6	37.2 (27.7–47.8)	96.6 (82.8–99.4)	52.2 (43.1–61.1)	66.9 (60.5–72.7)
Expert 1	94.2 (87.1–97.5)	89.7 (73.6–96.4)	93.0 (86.9–96.4)	91.9 (85.0–97.1)
Expert 2	88.4 (79.9–93.6)	65.5 (47.3–80.1)	82.6 (74.7–88.5)	76.9 (67.2–86.1)
Expert 3	89.5 (81.3–94.4)	72.4 (54.3–85.3)	85.2 (77.6–90.6)	81.0 (71.8–89.0)

Values are percentages (95% CI), including balanced accuracy. Sensitivity and specificity indicate agreement with the 86 reference-positive and 29 reference-negative classifications. Accuracy is overall binary agreement; balanced accuracy is their sensitivity–specificity average. Individual proportions use Wilson intervals; balanced accuracy uses 10,000 reference-stratified bootstrap resamples. PPV and NPV are in Supplementary File S3. The final reference was determined after access to the three experts’ classifications; comparisons with those experts are descriptive, not independent tests of diagnostic superiority.

**Table 4:** Direct AI–expert balanced agreement in the 115 treated participants.

AI Configuration	Expert 1	Expert 2	Expert 3
Gemini 3.5 Flash	47.8 (38.6–57.3)	42.3 (33.7–51.5)	48.5 (39.3–58.4)
Claude Sonnet 4.6	53.1 (43.1–62.9)	51.4 (41.0–61.7)	45.5 (35.4–55.7)
GPT-5.5	53.9 (47.9–59.5)	51.2 (44.3–57.6)	51.5 (44.7–57.6)
Grok 4.3	50.2 (45.7–55.7)	48.2 (44.2–52.8)	45.9 (42.9–48.2)
Kimi K2.6	60.8 (52.7–68.2)	55.4 (46.1–64.0)	64.9 (57.8–71.4)

Values are percentages (pointwise 95% CI). Each column uses the indicated expert’s independently recorded labels: category 1 is positive and categories 2–3 are not successful. Expert-positive/negative counts are 84/31, 86/29 and 85/30 for Experts 1–3, respectively. Balanced accuracy is the mean of the positive- and negative-class match proportions. Intervals use 10,000 expert-label-stratified participant bootstrap resamples, conditional on each expert’s labels and observed class distribution; they are not simultaneous intervals or superiority tests. Sparse and zero-match cells limit empirical bootstrap uncertainty. These subjective expert labels are not anatomical ground truth. Underlying counts and resampling details are in Supplementary Table S3.19.

**Table 5:** Exploratory three-category results in the five no-block image controls.

Rater	Successful, *n*	Patchy/Inadequate, *n*	Failed/No LA, *n*	Responses Asserting Any LA, *n*/5
Gemini 3.5 Flash	1	2	2	3/5
Claude Sonnet 4.6	2	3	0	5/5
GPT-5.5	0	1	4	1/5
Grok 4.3	5	0	0	5/5
Kimi K2.6	0	3	2	3/5
Five AI configurations combined	8	9	8	17/25
Expert 1	0	0	5	0/5
Expert 2	0	0	5	0/5
Expert 3	0	0	5	0/5
Senior-expert final reference	0	0	5	0/5

No block was performed, no needle was inserted and no local anaesthetic was injected in these controls. Only failed/no LA (category 3) was the expected response. Categories 1 and 2 both assert the presence of local anaesthetic. The 25 AI outputs are cross-classified within five cases and five AI configurations and must not be treated as 25 independent patient observations. This separate post hoc analysis is descriptive; the five controls do not estimate a general false-positive or safety-event rate. LA, local anaesthetic.

## Data Availability

De-identified participant-level analysis data underlying the reader-performance and longitudinal pain analyses, together with analysis code and derived machine-readable outputs, will be made available to qualified researchers on reasonable request to the corresponding author after publication, subject to Ethics Committee/institutional approval and an appropriate data-use agreement. The retained AI-derived dataset contains the recorded integer categories and derived analysis outputs; screenshots and any additional response text were not retained. Ultrasound images are not included in this general sharing commitment because of privacy, consent and governance constraints.

## Supplementary Materials

The following supporting information can be downloaded at: https://www.mdpi.com/article/10.3390/diagnostics16213555/s1, Supplementary File S1: AI configurations, image workflow and verbatim prompt. Supplementary File S2: STARD-AI and STROBE reporting checklists with explicit unmet items. Supplementary File S3: supplementary reader results, pain analyses and sensitivity checks. Supplementary File S4: protocol–registry–manuscript reconciliation. Figure S1: secondary reference-conditioned binary measures. Figure S2: exploratory adjusted pain contrasts. Reporting frameworks used in Supplementary File S2 are cited in references [21,22,23,24,25,26]; methodological sources for Supplementary File S3 are listed in references [35,36,37,38].

## Abbreviations

AI, artificial intelligence; ASA, American Society of Anesthesiologists; BMI, body mass index; CABG, coronary artery bypass grafting; CI, confidence interval; HADS, Hospital Anxiety and Depression Scale; ICU, intensive care unit; IQR, interquartile range; LA, local anaesthetic; LLM, large language model; MAR, missing at random; NPV, negative predictive value; NRS, numerical rating scale; PIFB, pecto-intercostal fascial plane block; PNG, Portable Network Graphics; PPV, positive predictive value; SD, standard deviation.
