# Supplementary material for: Five Multimodal AI Configurations Versus Anaesthesiologists for Pecto-Intercostal Block Classification in an Archived-Image Reader Study of a Prospective CABG Cohort
Source: Diagnostics (Basel). 2026 Oct 28;16(21):3555. doi: 10.3390/diagnostics16213555 (PMC13762392; doi:10.3390/diagnostics16213555)
Supplement: Supplementary file 1 [file diagnostics-16-03555-s001.zip › 03_Supplementary_S1_AI_Workflow.pdf]

Supplementary File S1

AI Configurations, Image-Query Workflow and Verbatim Prompt

S1.1. AI configurations evaluated

Five publicly accessible, general-purpose multimodal artificial intelligence (AI) configurations that accepted multiple images were selected pragmatically for accessibility and image-input capability, with the intention of sampling configurations the investigators regarded as commonly used. The number and identities of the configurations were fixed before any output was examined. The configuration labels below are the reporting labels derived from the investigators' June 2026 records; exact access dates, session logs and provider-side build identifiers were not available. The labels identify the consumer configurations selected by the investigators but do not independently verify the provider-side backend used for every query.

Table S1.1. Evaluated AI configuration labels and access conditions.

Reported AI configuration label	Provider	Access route	User-controlled settings	Assessment period
Gemini 3.5 Flash	Google	Standard free consumer web interface	Default response mode	June 2026
Claude Sonnet 4.6	Anthropic	Standard free consumer web interface	Default response mode	June 2026
GPT-5.5 in ChatGPT	OpenAI	Standard free consumer web interface	Default response mode	June 2026
Grok 4.3	xAI	Standard free consumer web interface	Default response mode	June 2026
Kimi K2.6	Moonshot AI	Standard free consumer web interface	Default response mode	June 2026

The investigators report that all AI configurations were used through free, non-subscription consumer accounts in their standard web interfaces and default response modes. No investigator-defined system instruction or custom instruction was added. The investigators did not select a non-default response mode. Provider-level system instructions, training data and provider-side build identifiers were not visible to the investigators. Provider data-use/model-training settings were not actively disabled. The investigators did not train, fine-tune, adapt or connect any model to a retrieval system for this study.

S1.2. Image inputs and case order

Although PIFB was bilateral, only the left side was recorded for the reader study. One researcher, distinct from the block operator and the four human readers, extracted 8–10 representative frames per participant and later performed the AI queries and recorded the first-response integers. This researcher had no access to postoperative pain data or to the expert or final-reference classifications. For treated participants, frames covered the pre-block anatomical view, needle advancement, local-anaesthetic spread and final post-injection appearance. Selection was judgement based: frames were not necessarily consecutive and there was no objective temporal-sampling rule or fixed number per stage. Case-level frame counts and per-stage annotations were not retained in the analysis dataset; no retrospective stage annotation or frame-count/needle-frame regression was performed. No-block sets came from left-sided anatomical recordings and lacked needle-advancement and injection frames. All sets were completed before any human or AI classification and were not subsequently altered. Only Portable Network Graphics (PNG) exports were uploaded. Filenames and image content contained no patient name, protocol number, date of birth, procedure date, hospital number or other direct identifier, and the source video files and any Digital Imaging and Communications in Medicine (DICOM) metadata or headers were not uploaded. No investigator-side cropping, resizing, brightness/contrast adjustment or analytical image normalisation was applied after frame export. The same de-identified PNG frames were supplied to all nine raters.

All AI configurations and human readers received the same image sets, the same frames in each set and the same fixed order of 120 participants. Image content was identical, but information available for the final reference differed: the senior expert reviewed Experts 1–3's labels before giving the final categories used in analysis. Within each case, frames were presented in the same chronological procedural order to every reader. Case order was not randomised. The treated cohort comprised 115 participants who received pecto-intercostal fascial plane block (PIFB). Five additional participants declined the block, underwent no needle insertion and received no local anaesthetic. The Ethics Committee-authorised study scope and these participants' separate written consent covered anatomical ultrasound recording and research/AI assessment of the de-identified images. The five no-block image sets were designated for inclusion in the common reader series before any human or AI output was observed; their separate exploratory control analysis was post hoc. The AI prompt explicitly warned that images without a performed block could be present. Human readers were likewise informed that no-block image sets could be present but were not told their number or positions.

S1.3. Query and response-handling procedure

The same researcher who selected the frames performed all 600 queries and recorded the first-response integers without access to postoperative pain data or to the expert or final-reference classifications. The researcher used the following unchanged first-response workflow:

1. Open a new conversation.

2. Upload the participant's 8–10 PNG frames in one message.

3. Submit the verbatim prompt in Section S1.4.

4. Record the integer returned in the first response in the study spreadsheet.

5. Delete that conversation from the visible account history.

6. Open a fresh conversation for the next participant.

No response was regenerated or re-prompted, and no study output was used to develop or select the prompt. No formal prompt-development or pretesting dataset was documented. According to the numerical record and investigator account, every first response supplied a valid category. Only the integers were retained, without raw text, screenshots or session logs, so response wording and universal first-response compliance cannot now be independently audited. Account memory and personalisation settings were not recorded or actively modified; new conversations do not prove absence of all provider-level contextual effects. One query per configuration and case precludes measuring within-configuration repeatability.

The device-preset instruction was informed by prior experience in other studies, not by outputs from the present evaluation.

S1.4. Standard prompt reproduced verbatim

The following prompt was used without alteration for every image set and every AI configuration. It is reproduced exactly, including the typographical error “Facial” in place of “Fascial” in the second paragraph.

You are an AI expert specializing in regional anesthesia and point-of-care ultrasound (POCUS).

I am uploading ultrasound (USG) images of Pectointercostal Facial Plane Block (PIFB) applied for sternotomy analgesia. The number of images may vary. Images without block may also be included for testing purposes. All images may have a block applied, or none may have one.

CRITICAL RULE: Do not consider any pre-set text, labels, or layers on the screen (e.g., “Carotid,” “Nerve,” “Vascular,” etc.). All uploaded images pertain to the pectointercostal fascial plane block. Do not refuse evaluation based on device presets.

Review the images, and if you believe a block was applied, find the post-injection images showing local anesthesia (LA) application and score the block success according to this scale.

EXACT SCORING REQUIREMENT: You MUST assign EXACTLY one of the following integers (1, 2, or 3). Do not use decimal numbers, fractions, or intermediate scores (e.g., do not give 1.5, 2.4, etc.). Select the nearest whole number that matches the criteria:

1 (Successful block): The local anesthetic solution is completely and clearly distributed within the target fascial planes, showing clear hydrodissection.

2 (Partial block): The local anesthetic solution is partially distributed within the fascial planes, showing leakage and/or poor spread to adjacent tissues.

3 (Unsuccessful block): Little or no local anesthetic solution is visible within the target fascial planes.

Evaluate

S1.5. Correspondence between AI-prompt and human-reader categories

Table S1.2. Correspondence between AI-prompt scores, human-reader categories and analysis labels.

Score	AI-prompt wording	Wording given to human readers	Analysis label
1	Successful block—local anaesthetic completely and clearly distributed within the target fascial planes, with clear hydrodissection	Successful—local anaesthetic distributed completely and clearly within the target fascial plane, with evident hydrodissection	Successful
2	Partial block—local anaesthetic partially distributed, with leakage and/or poor spread to adjacent tissues	Patchy/inadequate—local anaesthetic partly distributed within the plane, with leakage into adjacent tissue and/or poor spread	Patchy/inadequate
3	Unsuccessful block—little or no local anaesthetic visible within the target fascial planes	Failed/no local anaesthetic—little or no local anaesthetic visible in the target plane	Failed/no local anaesthetic

All human and AI outputs were recorded and analysed under the mapping shown in Table S1.2.

Categories 2 and 3 were combined only for the successful-versus-not-successful analysis in the 115 treated participants. They were not combined for the three-category analysis or for the five no-block controls: category 2 explicitly asserts partial local-anaesthetic spread, whereas category 3 is the expected response when no local anaesthetic was injected.

S1.6. Scope and reproducibility limits

This was a zero-shot, first-response, single-query evaluation of five specific consumer configurations. It did not assess prompt optimisation, repeated-query aggregation, temporal repeatability, in-context examples, fine-tuning, task-specific models or clinician–AI collaboration. Proprietary model updates, provider-level instructions, unrecorded account-level memory/personalisation settings and other defaults may limit exact external replication. In the quoted prompt, USG refers to ultrasonography. The effect of the verbatim prompt's "Facial" typo and non-standard "USG" abbreviation was not tested. Exact interface labels cannot now be authenticated from screenshots.
