# Supplementary material for: Five Multimodal AI Configurations Versus Anaesthesiologists for Pecto-Intercostal Block Classification in an Archived-Image Reader Study of a Prospective CABG Cohort
Source: Diagnostics (Basel). 2026 Oct 28;16(21):3555. doi: 10.3390/diagnostics16213555 (PMC13762392; doi:10.3390/diagnostics16213555)
Supplement: Supplementary file 1 [file diagnostics-16-03555-s001.zip › 04_Supplementary_S2_Reporting_Checklists.pdf]

Supplementary File S2 Reporting Checklists

STARD-AI is the principal reporting framework for the archived-image AI reader comparison, and STROBE covers the prospective cohort. Relevant STARD 2015, CLAIM 2024, TRIPOD-LLM and GRAITE-USRA considerations were also consulted. These frameworks were applied during reporting, not as prospective protocol-design standards.

The reader measures describe agreement with a senior anaesthesiologist's final subjective reference, determined after reviewing the three experts' classifications. Use of STARD-AI does not establish anatomical diagnostic truth, prospective prespecification, or clinical readiness. The principal binary cohort contains 115 treated participants; the five no-block image sets are a separate exploratory control analysis.

Met means that the reporting information is supplied, not that the study is free from bias. Partially met identifies reported components and remaining limitations. Not met identifies a missing requirement or record; stating that it is absent does not retrospectively fulfil it. Not applicable is reserved for requirements outside this design. Not performed identifies an optional analysis that was not undertaken. Locations refer to the revised manuscript and named supplementary sections.

STROBE checklist for the prospective cohort component

von Elm E, Altman DG, Egger M, et al. The Strengthening the Reporting of Observational Studies in Epidemiology (STROBE) statement. Lancet. 2007;370:1453–1457. doi:10.1016/S0140-6736(07)61602-X.

Item	Status	Location	What is reported and what remains unmet
1a. Identify design in title/abstract	Met	Title; Abstract; §2.1	The prospective cohort and subsequent archived-image reader study are identified.
1b. Balanced abstract	Met	Abstract	The descriptive design, expert-informed final reference, principal results and restricted June 2026 scope are stated. Pain is secondary and exploratory. No reproducible a priori power calculation was available for the present binary reader comparison, and raw AI outputs were not retained.
2. Background/rationale	Met	§1	Clinical context and the rationale for comparing general-purpose multimodal configurations with anaesthesiologists are supplied.
3. Objectives/hypotheses	Partially met	§1; §§2.2, 2.8; S4 outcome hierarchy	Current objectives are distinguished from the original pain-first protocol and earlier registry. The signed April version 4 makes each LLM's three-category balanced accuracy primary and pain associations secondary. The present treated-cohort binary comparison is narrower than that protocol endpoint; retrospective registration and post hoc inferential analyses are identified.
4. Key study-design elements	Met	§§2.1–2.3, 2.5–2.8	Prospective collection, later image reading, single-centre sampling, reference process, treated cohort and exploratory controls are described.
5. Setting and relevant dates	Partially met	§§2.1–2.3, 2.7; Table S3.20; S4 registration and study dates	Centre, recruitment period, final imaging date, clinical data-completion date, June reading month and monthly study enrolment counts (Table S3.20) are reported. Exact reading dates and actual pain-assessment timestamps remain unavailable.
6a. Eligibility, selection and follow-up	Met	§§2.3–2.5, 2.8; §3.1; Figure 1	Eligibility, consecutive invitation on operator-available days, imaging and scheduled clinical follow-up are described. The unlogged population on other days is disclosed under non-participation.
6b. Matched-study criteria	Not applicable	§2.1	This was not a matched cohort. Comparing readers on the same images is a paired measurement design, not participant matching.

Item	Status	Location	What is reported and what remains unmet
7. Variables and definitions	Met	§§2.4, 2.6, 2.8, 2.10; S1 category definitions; S3 analysis definitions	Reference categories, positive class, NRS outcomes, nominal time points, covariates and rescue-receipt interval are defined. The qualitative spread categories have no validated quantitative cut-off.
8. Data sources and measurement	Partially met	§§2.4–2.8; S1 image and query workflow; S3 data coding and missingness	Sources, equipment, frame construction and classification procedures are described. Raw AI text, exact assessment times, stage-specific frame metadata and pain-assessor standardisation records are unavailable; only categorical reader outputs are retained.
9. Bias	Met	§§2.3, 2.5–2.7; §4	Reported safeguards include common images, independent assessments by Experts 1–3, blinded AI querying and selection before ratings. The senior expert viewed the three experts' labels before finalising the reference; comparisons of those experts with this reference are not independent accuracy assessments. Disclosure does not remove reference dependence, selection, frame-curation, fixed-order, platform or clinical confounding limitations.
10. Study size	Partially met	§§2.2, 2.9; Table 3; S4 enrolment amendment	Approved cohort targets and achieved reader precision are reported. The original pilot calculation and signed April assertion of power above 90% lack the assumptions needed for reconstruction and do not provide a reproducible a priori justification for the present binary comparison. No equivalence or non-inferiority margin was specified.
11. Handling quantitative variables	Met	§§2.8, 2.10; Table 1; S3 model specification and subgroup error summaries	Summary measures, continuous covariate handling, nominal categorical time and post hoc BMI-band boundaries are specified. The ordinal NRS and its model estimand are distinguished from Gaussian NRS-point contrasts.
12a. Statistical methods/confounding	Met	§2.10; S3 reader-analysis methods and pain-model specification	Paired reader methods, pointwise confidence intervals, clinically chosen pain covariates and model assumptions are described. Paired reader p values are not reported. Unavailable rescue timing prevents time-varying medication adjustment; residual confounding remains.
12b. Subgroup/interaction methods	Met	§2.10; S3 sex and BMI error summaries; S3 pain-model specification	Reference-group-by-time interactions and descriptive error summaries by sex and post hoc BMI bands are identified. No powered subgroup fairness or effect-modification claim is made.
12c. Missing-data methods	Partially met	§§2.8, 2.10; §3.1; S3 missingness and delta-adjusted ordinal sensitivity	Available-observation assumptions and selected delta-adjusted sensitivity scenarios are described. These cannot identify the true missing scores or prove missing at random. The two never-extubated participants have no self-report scores and are excluded from pain modelling and imputation.
12d. Loss to follow-up	Met	§§2.8, 3.1; Figure 1; S3 pain availability	Unavailable assessments during ventilation and the two never-extubated deaths are distinguished from a lost-to-follow-up cohort. Available observations and reasons for non-observation are reported at each nominal visit.
12e. Sensitivity analyses	Met	§2.10; S3 expert-majority comparison, ordinal pain models, Gaussian comparisons and delta-adjusted sensitivity	Post hoc sensitivity methods and their assumptions are specified. Identical majority labels do not provide independent reference validation; selected missingness shifts do not establish that all departures from missing at random have been addressed.

Item	Status	Location	What is reported and what remains unmet
13a. Participant numbers at each stage	Partially met	§§2.3, 3.1; Figure 1	Flow is supplied for all 120 enrolled participants, 115 treated participants, five controls and available pain observations. The total potentially eligible population on non-recruitment days was not logged.
13b. Reasons for non-participation	Partially met	§2.3; §4; Figure 1; Table S3.20	No invited eligible patient on recruitment days declined study participation; five declined the block but consented to imaging. Numbers and reasons for non-participation on other days cannot be determined. The work-organisation explanation and monthly enrolment series are provided in §2.3 and Table S3.20.
13c. Flow diagram	Met	Figure 1; §3.1	The diagram distinguishes enrolled, treated and no-block groups and the left-sided image-reading task despite bilateral treatment.
14a. Participant characteristics	Met	§3.1; Table 1; S3 baseline characteristics by reference group	Treated-cohort demographic and perioperative characteristics are supplied with denominators. Race and ethnicity were not collected; the cohort was selected and is not population representative.
14b. Missing values by variable	Met	Table 1 and notes; §3.1; S3 per-variable missingness and pain availability	Variable-specific available/unavailable counts and coding conventions are stated. Diabetes and smoking use investigator-confirmed present/absent coding. Blanks in the other presence-coded fields are not represented as individually verified negative observations; complete recorded BMI does not imply complete height and weight fields.
14c. Follow-up time	Partially met	§§2.1, 2.8, 3.1; Table 1; S3 pain availability	Scheduled 48-hour pain follow-up, clinical data completion and index-admission stay summaries are given. Actual pain-assessment timestamps and tolerance windows were not retained; nominal labels cannot be converted into observed elapsed time after extubation.
15. Outcome data	Met	§§3.2–3.6; Tables 2–5; Figures 1–2; S3 pain and rescue-receipt results	Reader outcomes, denominators and observed pain outcomes are supplied. The uncollected mobilisation outcome and unreconstructible registered 24-hour analgesic consumption are explicitly disclosed rather than reported as results.
16a. Unadjusted/adjusted estimates and precision	Met	§§3.2–3.6; Tables 2–5; Figure 2; S3 pain estimates and sensitivity results	Reader and pain estimates include uncertainty and analysis denominators. Covariate adjustment and exploratory multiplicity handling are specified; pointwise intervals are not described as simultaneous intervals.
16b. Category boundaries	Met	§§2.6, 2.8, 2.10; S1 category mapping; S3 subgroup error definitions	The three reader categories, binary collapse and post hoc BMI bands are explicit. Qualitative spread definitions were not retrospectively converted into invented quantitative thresholds.
16c. Convert relative to absolute risk	Not applicable	§§2.8, 2.10	The study does not estimate causal exposure-related event risks requiring conversion over follow-up. Reader proportions are reported directly; ordinal pain odds ratios are not event-risk reductions.
17. Other analyses	Met	§§3.3–3.6; S3 expert-majority comparison, no-block responses, subgroup errors and pain sensitivities	Other analyses and their post hoc status are disclosed. Fixed-interval frame experiments and repeated AI queries were not performed.

Item	Status	Location	What is reported and what remains unmet
18. Key results	Met	§4; §5	The principal reader-agreement findings and secondary pain association are related to the study objectives without using pain to validate the reference.
19. Limitations	Met	§4; S1 limitations; S4 evidence limitations	Reference subjectivity, unavailable records, selection, precision, curated inputs, platform version uncertainty, missingness and confounding are described, including implications for interpretation.
20. Interpretation	Met	§4; §5	Conclusions remain conditional on the tested reference, cases and June 2026 configurations. Majority agreement, post hoc pain associations and control responses are not treated as external validation or causal effects.
21. Generalisability	Met	Abstract; §4; §5	Single centre/operator/system, ASA III isolated-CABG cohort, selected recruitment days, free consumer access and one assessment period are identified as limits to transferability.
22. Funding and funder role	Met	Funding; Conflicts of Interest; §2.11; Acknowledgments	No external funding or evaluated-provider sponsorship is declared; post-study AI assistance and author responsibility are separately disclosed.

STARD-AI checklist for the archived-image reader comparison

Sounderajah V, Guni A, Liu X, et al. The STARD-AI reporting guideline for diagnostic accuracy studies using artificial intelligence. Nat. Med. 2025;31:3283–3289. doi:10.1038/s41591-025-03953-8.

Item	Status	Location	What is reported and what remains unmet
1. AI diagnostic-performance identification	Met for reader-study identification	Title; Abstract; §2.1	The report identifies an AI reader comparison and its subjective reference. It deliberately avoids presenting the task as anatomical diagnostic accuracy.
2. Structured abstract	Met	Abstract	The structured summary states the design, principal metric, expert-informed final reference, results, absence of a reproducible power calculation for the present binary comparison and major auditability/generalisation limits.
3. Background, intended use and workflow	Met	§1; §§2.5–2.7; §4	The archived-image interpretation task and its distinction from real-time guidance or autonomous clinical use are explained. No implemented clinical deployment is claimed.
4. Objectives/hypotheses	Partially met	§1; §§2.2, 2.8; S4 outcome hierarchy	The original protocol and earlier registry were pain-first. The signed April version 4 specifies each LLM’s three-category balanced accuracy as primary, with pain associations and comparisons with individual experts secondary. Current binary reader comparisons are described as a narrower, descriptive adaptation; the later registry update does not establish prospective registration or prespecification of added inferential analyses.
5. Prospective/retrospective data timing	Met	§§2.1–2.2, 2.7	Prospective recruitment, imaging and follow-up are distinguished from June 2026 archived-image readings and retrospective registration.

Item	Status	Location	What is reported and what remains unmet
6. Ethics approval	Met for approval reporting	§2.2; Institutional Review Board and Consent statements; S4 ethics-document reconciliation; confidential editorial documents	Approval identity, dates and consent are reported. The 13 August decision, 14 August transmittal, 6 May decision, original August electronic documents and investigator-signed April version 4 protocol/application scans are supplied to the editor. The investigators identify the April scans as the revised submission accompanying the LLM clarification and sample enlargement approved on 6 May. Investigator-signed forms are distinguished from the committee’s decision; independent signature or archive authentication is not claimed.
7. Participant- and data-level eligibility	Met	§§2.3, 2.5; §3.1; Figure 1	Clinical eligibility, treated/no-block status and common left-sided image-set construction are defined. No objective temporal frame-selection rule existed.
8. Basis for identifying eligible participants/data	Met	§§2.3, 2.5	Eligible patients were consecutively invited on operator-available days; image sets were constructed before any classification. Patients on other days were not logged.
9. Setting, locations and dates	Partially met	§§2.1–2.3, 2.7; S1 configuration records; S4 dates; Table S3.20	Centre, clinical dates, June assessment period and monthly study enrolment counts (Table S3.20) are reported. Exact query-day ranges and individual session dates remain unavailable.
10. Sampling	Partially met	§§2.3, 2.5, 2.7; §4	Operator-available recruitment, pragmatic AI selection and judgement-based frame sampling are described. The non-recruitment-day denominator and objective time-sampling/frame-stage data are missing.
11. Data source and purpose	Met	§§2.1, 2.4–2.5	Images were collected prospectively in the study cohort and assessed later. The same de-identified frames were used for all readers; the conducted block and separate no-block recordings are distinguished.
12. Annotators and annotation context	Partially met	§2.6; S1 category definitions; S3 expert vote patterns	Human-reader number, experience, common definitions, independent classifications by Experts 1–3 and the senior expert’s review of their labels before determining the final reference are reported. No calibration exercise or validated quantitative rubric was used. The report does not claim a timestamped audit of the reading sequence.
13. Capture devices and AI software/model versions	Partially met	§§2.4, 2.7; S1 configuration and input details	Ultrasound device/transducer, nominal frequency, image format, reported consumer labels and access tier are given. Exact displayed interface evidence, backend build identifiers and per-query versions are not available; the version-verification component is not met.
14. Acquisition and preprocessing	Partially met	§2.5; S1 image-set construction	Left-sided video acquisition, representative frames, procedural stages, de-identification and absence of investigator post-export image adjustments are described. Per-case/stage counts, objective sampling times and provider-side processing are not documented.
15a. Index-assessment replication detail	Met for conducted workflow	§2.7; S1 verbatim prompt and query workflow	The unchanged prompt, images per query, single first-response rule, fixed case order, default settings and new-chat process are reported. Exact backend/session replication remains limited by missing dates, build identifiers and raw records; those omissions are listed separately.
15b. Development/training/validation/testing split	Not applicable	§2.7; S1 configuration scope	Investigators did not develop, train, fine-tune or select a fitted model on this dataset; no investigator training split existed. Proprietary provider training-data provenance remains unavailable under item 29.

Item	Status	Location	What is reported and what remains unmet
15c. Index categories/cutoffs	Met	§§2.6–2.8; S1 verbatim prompt and category mapping	Three forced categories and the treated-cohort binary grouping are defined. No quantitative validated anatomical cut-off or abstention option was used.
15d. End user and expertise	Partially met	§§2.6–2.7; §4	Human comparators' clinical experience and the separate querying researcher are described. No deployed clinician–AI workflow, user-performance study or standardised display/interaction assessment was performed.
16a. Reference-standard detail	Met	§2.6; §3.3; S3 expert vote patterns	The senior expert's experience, rubric and review of the three experts' labels before determining the final reference are described. Reader–reference analyses use the final decisions. This subjective reference is not independent of the three experts' classifications.
16b. Reference rationale	Met	§2.6; §4	The pragmatic rationale and absence of a feasible independent anatomical criterion are stated. The rationale does not establish external validity.
16c. Reference categories/cutoffs	Met	§2.6; §3.2; Table 2	Reference categories and their counts are reported; none of the 115 treated cases had reference category 3. No validated numerical threshold was specified or reconstructed.
17a. Information available to AI/index readers	Met	§§2.5–2.7; S1 inputs and querying	Common de-identified images, fixed prompt/order and absence of participant-level clinical information are described. The query researcher had no access to the expert/reference scores; human-reader blinding is reported separately.
17b. Information available to reference	Met	§2.6	The senior expert saw the same images and category definitions and knew that controls could be present, without knowing their number or positions. The three experts' classifications were available before the final reference decision; AI outputs and clinical outcomes remained masked.
18. Accuracy-analysis methods	Met for conditional reader measures	§2.10; S3 reader-analysis methods	Reference-conditional measures, paired resampling and pointwise confidence intervals are specified; paired p values are not reported. These procedures do not remove dependence between the final reference and experts whose labels were reviewed. Results are descriptive agreement comparisons, not independent anatomical diagnostic-accuracy validation.
19. Indeterminate/uninterp retable outputs	Partially met	§§2.7, 2.10; S1 response handling	The conducted task forced three categories and the retained record contains a valid category for every query. Absence of refusals or other original wording cannot be independently verified because raw outputs were deleted; no abstention or uninterpretable-image category was available.
20. Missing index/reference results	Met for retained classifications	§§2.10, 3.1; S3 reader-data completeness	All 1,080 case-level classifications are present in the retained categorical record, including all nine readers. Complete recorded categories do not demonstrate complete retention of original AI responses.
21. Variability analyses	Partially met	§§2.10, 3.2–3.4; S3 agreement summaries and paired comparisons	Between-reader agreement, paired differences, observed agreement and exploratory subgroup errors are described. One AI query per case precludes within-configuration repeatability or temporal-drift analysis.

Item	Status	Location	What is reported and what remains unmet
22. Intended sample size	Partially met	§§2.2, 2.9; S4 enrolment amendment	The approved cohort size and achieved precision are given. The original pilot-based calculation and signed April assertion of power above 90% cannot be reconstructed from the supplied assumptions and do not justify power for the current 115-participant binary comparison.
23. Error, bias and fairness analyses	Partially met	§§3.2–3.5; §4; S3 confusion matrices and sex/BMI error summaries	Categorical error patterns, separate control responses and exploratory sex/BMI strata are reported. Small imbalanced strata do not establish fairness or equivalence; race/ethnicity data and a prespecified fairness assessment are absent.
24. Participant/data flow	Partially met	§§2.3, 3.1; Figure 1; Table S3.20	Enrolled, treated, no-block and recorded-reading flow and monthly study enrolment (Table S3.20) are supplied. The number of eligible patients on non-recruitment days cannot be supplied.
25. Demographic, clinical and technical characteristics	Partially met	§§2.4–2.5, 3.1; Table 1; S3 baseline characteristics and missingness	Demographic/perioperative summaries, equipment and missingness/coding are reported. Race/ethnicity, standardised human display conditions and case-level frame-stage metadata were not collected.
26a. Target-condition severity	Not applicable	§§2.6, 3.2; Table 2	No disease-severity continuum was evaluated. The qualitative spread-category distribution is reported without treating it as a validated severity measure.
26b. Alternative diagnoses	Not applicable	§2.8	This was a procedural image-classification task, not differential diagnosis of a target disease.
27. Interval/interventions	Partially met	§§2.1, 2.5–2.7	All raters assessed the same archived sets after clinical follow-up, without altering patient care or images between readings. June is known, but exact reader/query dates and intervals between individual ratings are unavailable.
28. Representation of intended-use distribution	Partially met	§§2.3, 2.5; §4	The selected ASA III isolated-CABG cohort, one centre/operator/system and free consumer workflow are described. No sampling comparison establishes representation of a broader clinical intended-use population.
29. Independence/differences from model-development data	Not met	§2.7; S1 configuration limitations; §4	Investigators undertook no study-specific model training, but proprietary training/validation data and potential overlap cannot be independently inspected. Separation from provider development data is not established.
30. Cross-tabulation	Met	§§3.2–3.5; Tables 2–5; S3 treated and all-120 confusion matrices	Reader/reference cross-tabulations and the separate control distributions are supplied. The constructed all-120 mixture is not treated as failed-injection diagnostic performance.
31. Accuracy and precision	Met for conditional measures	§§3.2–3.4; Tables 2–3; Figure 2; S3 paired differences	Estimates, denominators and confidence intervals are reported. Binary balanced accuracy is the principal descriptive measure of this report, distinguished from the signed April three-category endpoint. Intervals are pointwise; estimates depend on the final reference, which was determined after access to the three experts' classifications.
32. Adverse events from index/reference assessment	Not applicable	§§2.1, 2.7–2.8; §3.1	Archived-image assessments occurred after care and did not constitute a new clinical intervention. Clinical cohort complications are reported separately; this does not resolve the distinct platform-governance limitations.

Item	Status	Location	What is reported and what remains unmet
33. Limitations, bias, uncertainty and generalisability	Met	Abstract; §4; S1 limitations; S4 evidence limitations	Design, reference, sampling, frame selection, missing records, imprecision, platform changes and exploratory clinical assumptions are discussed without claiming they were corrected by reanalysis.
34. Practice implications/intended role	Met	§4; §5	Interpretation is limited to the tested reader task; autonomous use, real-time guidance, task-adapted models and clinician–AI collaboration were not validated.
35. Ethics and fairness	Partially met	§2.2; Consent statement; §4; S1 governance; S3 subgroup errors; S4 ethics reconciliation	Consent and de-identification are reported, with default consumer data-use settings and limited documentation acknowledged. Provider retention/deletion, comprehensive institutional platform governance and demographic fairness are not established.
36. Registration	Partially met; prospective registration not met	§2.2; Institutional Review Board statement; S4 registration and outcome hierarchy	NCT07581678 and submission/posting dates are reported. Registration began after recruitment. The original protocol, signed April outcome revision, earlier registry and retrospective September update are distinguished. Neither the current registry record nor its later update establishes prospective registration of the present binary endpoint or added analyses.
37. Protocol access	Met for confidential editorial access; not public access	§2.2; S4 protocol and amendment reconciliation; confidential editorial document index	The original August protocol/application are supplied as electronic copies and the revised April version 4 protocol/application as investigator-signed scans, alongside the 13 August, 14 August and 6 May decision/transmittal documents. Source originals remain unchanged; investigator signatures are not treated as independent committee-archive authentication. S4 describes their contents and differences; full source documents are confidential editorial material.
38. Funding and funder role	Met	Funding; Conflicts of Interest	No external funding or study-specific support from evaluated providers is declared.
39. Commercial interests	Met	Conflicts of Interest; §2.11; Acknowledgments	Provider involvement and competing interests are disclosed; manuscript-preparation assistance is separated from the evaluated AI configurations.
40a. Dataset/code/output availability and restrictions	Partially met	Data Availability statement; S1; S3 analysis provenance; confidential classification table	Controlled numerical-data/code sharing and its conditions are described; editor/reviewer case-level categories permit aggregate recalculation. Images are excluded from the general sharing commitment, and no publicly reproducible raw-session archive exists.
40b. Storage/auditability of AI outputs	Not met	Abstract; §2.7; Data Availability statement; S1 response retention	Only the first recorded integer category was retained. Raw response text, screenshots, session logs, exact dates and backend identifiers are unavailable. The numerical table cannot authenticate original wording, transcription fidelity or universal first-response compliance.

Registration and protocol

TRIPOD-LLM 14c–14d; GRAITE-USRA 37–38. Protocol documents are supplied confidentially; prospective registration remains not met. S4 distinguishes the original August electronic documents, investigator-signed April version 4 protocol/application, separate committee decisions and registry history. The signed April protocol makes each LLM’s three-category balanced accuracy primary, unlike the original pain-first protocol and earlier registry. The present 115-participant binary comparison is narrower than that planned endpoint; the later registry update does not establish prospective registration or prespecification of analyses added during revision. Full source documents are available to the editor, not publicly.

Study size

STARD 2015 18; GRAITE-USRA 19. Partially met. Approved size and achieved precision are reported in §2.9; no a priori reader-comparison calculation is reconstructed.

Model versions and retained outputs

TRIPOD-LLM 6a, 6d; CLAIM 22. Partially met for configuration description; TRIPOD-LLM 6d not met. Reported labels and June access context are available, but raw outputs and exact build/session evidence are absent (§2.7; S1).

Error and fairness analysis

TRIPOD-LLM 19a, 19c. Partially met. S3 provides descriptive sex/BMI errors with uncertainty; this selected small cohort does not establish fairness, and race/ethnicity were not collected.

Reasons for non-participation

STROBE 13b. Partially met. Recruitment-day flow and monthly study enrolment are reported; eligible patients on other days were not logged (§2.3; Figure 1; Supplementary Table S3.20).

Missing values per variable

STROBE 14b. Met for reporting available counts and coding. Table 1 and S3 identify variable-specific availability and coding. Diabetes and smoking use investigator-confirmed present/absent coding; original blanks in the other presence-coded fields are not recast as individually verified negatives.

Missing-data assumptions

CLAIM 12; STROBE 12c–12d. Partially met. S3 reports ordinal and selected delta sensitivity assumptions. These do not establish MAR, recover actual timestamps or create assessable NRS in the two never-extubated participants.

Prompt development

TRIPOD-LLM 9a–9b. Development disclosure met; validation not performed. §2.7 and S1 state that the prompt was unchanged and no formal prompt-development/pretesting dataset was documented. The typo effect was not tested; no prompt validation is implied.

Replication detail

TRIPOD-LLM 6c. Met for the described prompt and workflow. S1 retains the verbatim prompt and conducted procedure. Availability of these details does not remedy absent raw transcripts or backend/version evidence.

Explainability

CLAIM 31. Not performed. The study did not evaluate model explanations or access model internals; no explanation-based reliability claim is made.
