# Supplementary material for: Five Multimodal AI Configurations Versus Anaesthesiologists for Pecto-Intercostal Block Classification in an Archived-Image Reader Study of a Prospective CABG Cohort
Source: Diagnostics (Basel). 2026 Oct 28;16(21):3555. doi: 10.3390/diagnostics16213555 (PMC13762392; doi:10.3390/diagnostics16213555)
Supplement: Supplementary file 1 [file diagnostics-16-03555-s001.zip › 05_Supplementary_S3_Statistical_Appendix.pdf]

Supplementary File S3 Statistical Appendix

S3.1 Analysis scope and provenance

The principal descriptive reader comparison uses 115 treated participants (86 reference-successful, 29 reference-patchy/inadequate). Final-reference analyses use the senior expert's final classifications, determined after review of the three experts' labels. Direct AI–individual-expert comparisons use each expert's labels instead (main Table 4; Table S3.19). Five no-block image controls are separate. No treated participant had reference category 3. Classifications from all 120 image sets may be tabulated for audit but do not estimate recognition of actual failed injections. The study was retrospectively registered; outcome-history reconciliation is in Supplementary File S4. Balanced accuracy (BA) is the mean of reference-conditioned sensitivity and specificity.

Table S3.1 Analysis populations

Analysis	Population	Denominator
Treated-cohort three-category reader performance	PIFB participants	115
All-case descriptive matrices and group-agreement audit	All consented image sets	120
Binary successful-versus-not-successful performance	Participants who received PIFB	115
Exploratory no-block control analysis	Participants who declined PIFB and received no injection	5
Longitudinal resting and coughing NRS models	Participants with at least one analysed pain measurement	113 participants; 331 observations

S3.2 Binary reader data

Table S3.2 Underlying treated-cohort binary counts

Rater	True positive	False negative	False positive	True negative
Gemini 3.5 Flash	56	30	22	7
Claude Sonnet 4.6	33	53	13	16
GPT-5.5	12	74	2	27
Grok 4.3	80	6	28	1
Kimi K2.6	32	54	1	28
Expert 1	81	5	3	26
Expert 2	76	10	10	19
Expert 3	77	9	8	21

Table S3.3 Predictive values at the observed reference prevalence

Reader	PPV % (95% CI)	NPV % (95% CI)
Gemini 3.5 Flash	71.8 (61.0–80.6)	18.9 (9.5–34.2)
Claude Sonnet 4.6	71.7 (57.5–82.7)	23.2 (14.8–34.4)
GPT-5.5	85.7 (60.1–96.0)	26.7 (19.1–36.1)
Grok 4.3	74.1 (65.1–81.4)	14.3 (2.6–51.3)
Kimi K2.6	97.0 (84.7–99.5)	34.1 (24.8–44.9)
Expert 1	96.4 (90.0–98.8)	83.9 (67.4–92.9)
Expert 2	88.4 (79.9–93.6)	65.5 (47.3–80.1)
Expert 3	90.6 (82.5–95.2)	70.0 (52.1–83.3)

PPV, positive predictive value; NPV, negative predictive value. Wilson intervals use the corresponding predicted-class denominator. These estimates depend on the reference-positive fraction 86/115, not an external disease prevalence. Figure S1 displays secondary measures.

S3.3 All case descriptive classification counts

Table S3.4 Three-category matrices for the constructed all 120 sample

Rater	Reference category (n)	Predicted 1: successful	Predicted 2: patchy/inadequate	Predicted 3: failed/no LA
Gemini 3.5 Flash	1: Successful (86)	56	28	2
Gemini 3.5 Flash	2: Patchy/inadequate (29)	22	7	0
Gemini 3.5 Flash	3: Failed/no local anaesthetic (5)	1	2	2
Claude Sonnet 4.6	1: Successful (86)	33	53	0
Claude Sonnet 4.6	2: Patchy/inadequate (29)	13	16	0
Claude Sonnet 4.6	3: Failed/no local anaesthetic (5)	2	3	0
GPT-5.5	1: Successful (86)	12	63	11
GPT-5.5	2: Patchy/inadequate (29)	2	24	3
GPT-5.5	3: Failed/no local anaesthetic (5)	0	1	4
Grok 4.3	1: Successful (86)	80	6	0
Grok 4.3	2: Patchy/inadequate (29)	28	1	0
Grok 4.3	3: Failed/no local anaesthetic (5)	5	0	0
Kimi K2.6	1: Successful (86)	32	54	0
Kimi K2.6	2: Patchy/inadequate (29)	1	28	0
Kimi K2.6	3: Failed/no local anaesthetic (5)	0	3	2
Expert 1	1: Successful (86)	81	5	0
Expert 1	2: Patchy/inadequate (29)	3	26	0
Expert 1	3: Failed/no local anaesthetic (5)	0	0	5
Expert 2	1: Successful (86)	76	10	0
Expert 2	2: Patchy/inadequate (29)	10	19	0
Expert 2	3: Failed/no local anaesthetic (5)	0	0	5
Expert 3	1: Successful (86)	77	9	0
Expert 3	2: Patchy/inadequate (29)	8	21	0

Rater	Reference category (n)	Predicted 1: successful	Predicted 2: patchy/inadequate	Predicted 3: failed/no LA
Expert 3	3: Failed/no local anaesthetic (5)	0	0	5

LA denotes local anaesthetic. Categories 1, 2, 3 denote successful, patchy/inadequate and failed/no LA. The five reference-category 3 cases are no-block controls, not failed attempted blocks. The all-120 matrices are descriptive and are not used to estimate recognition of genuine procedural failures.

Table S3.5 All 120 descriptive weighted kappa

Rater	Linear-weighted κ (95% CI)
Gemini 3.5 Flash	0.040 (−0.151 to 0.230)
Claude Sonnet 4.6	−0.040 (−0.172 to 0.095)
GPT-5.5	0.103 (0.021 to 0.191)
Grok 4.3	−0.047 (−0.129 to 0.052)
Kimi K2.6	0.261 (0.156 to 0.376)
Expert 1	0.865 (0.755 to 0.949)
Expert 2	0.657 (0.488 to 0.789)
Expert 3	0.711 (0.553 to 0.835)

These coefficients depend on including the structurally different controls; the main Table 2 reports only 115 treated cases. Whole-participant bootstrap resamples preserve all readings from each sampled case.

S3.4 Paired reader differences

Table S3.6 AI minus expert balanced accuracy in percentage points

AI configuration	Expert	BA difference pp (95% CI)
Gemini 3.5 Flash	Expert 1	-47.3 (-58.2–-35.8)
Gemini 3.5 Flash	Expert 2	-32.3 (-45.0–-19.1)
Gemini 3.5 Flash	Expert 3	-36.3 (-49.6–-22.5)
Claude Sonnet 4.6	Expert 1	-45.1 (-55.5–-35.3)
Claude Sonnet 4.6	Expert 2	-30.2 (-43.4–-16.4)
Claude Sonnet 4.6	Expert 3	-34.2 (-48.6–-19.3)
GPT-5.5	Expert 1	-38.4 (-46.5–-29.7)
GPT-5.5	Expert 2	-23.4 (-34.4–-12.5)
GPT-5.5	Expert 3	-27.4 (-38.9–-16.5)
Grok 4.3	Expert 1	-43.7 (-50.1–-36.2)
Grok 4.3	Expert 2	-28.7 (-38.5–-18.9)
Grok 4.3	Expert 3	-32.7 (-42.6–-21.3)
Kimi K2.6	Expert 1	-25.0 (-33.7–-15.8)
Kimi K2.6	Expert 2	-10.1 (-21.6–1.5)
Kimi K2.6	Expert 3	-14.1 (-23.3–-4.8)

Each comparison uses the same 115 treated participants. Intervals are pointwise 95% intervals from 10,000 reference-stratified participant resamples. Paired p values are not reported. Seeds and exact unrounded results are retained with the code. The final reference is treated as fixed in these calculations. Because it was determined with access to the experts' classifications, these paired differences and intervals cannot establish independent expert superiority or correct for reference construction.

S3.5 Expert pattern and group agreement

Table S3.7 Expert vote patterns across 120 image sets

Senior-expert final reference	Expert 1	Expert 2	Expert 3	Image sets, n
1	1	1	1	62
1	1	1	2	9
1	1	2	1	10
1	2	1	1	5
2	1	2	2	3
2	2	1	2	10
2	2	2	1	8
2	2	2	2	8
3	3	3	3	5

Seventy-five cases were unanimous and 45 had exactly one expert dissenting. The 8, 20 and 17 discordances did not overlap. The majority and final reference labels were identical; substitution therefore cannot independently validate the reference. The senior expert had access to these expert labels before finalising the reference, so agreement with the majority is not external corroboration. A confidential review-only workbook supplies all 1,080 integers without clinical identifiers; its row order is not study-reading order.

Table S3.8 Within-group agreement

Rater group and classification	n	Fleiss κ (95% CI)	Complete agreement	Mean pairwise observed agreement
Within experts, three categories; treated only	115	0.324 (0.193 to 0.451)	70/115	73.9%
Within AI, three categories; treated only	115	-0.101 (-0.133 to -0.067)	1/115	42.0%
Within experts, binary; treated only	115	0.324 (0.193 to 0.451)	70/115	73.9%
Within AI, binary; treated only	115	-0.104 (-0.138 to -0.067)	1/115	44.9%
Within experts, three categories; treated + no-block	120	0.424 (0.283 to 0.551)	75/120	75.0%
Within AI, three categories; treated + no-block	120	-0.090 (-0.124 to -0.054)	1/120	41.4%

Fleiss κ intervals are pointwise percentile intervals from 10,000 ordinary participant-level bootstrap resamples (seed 20260903), retaining all readers' ratings for each sampled participant together. Three-category and binary coding are shown separately; all-120 estimates describe a mixture containing five no-block controls. In the binary treated sample, fixed-marginal column permutations gave mean null Fleiss κ = −0.084 for AI and approximately 0 for experts, compared with observed −0.104 and 0.324. This illustrates heterogeneous reader marginal tendencies; no additional benchmark p value or within-configuration repeatability claim is made.

Nominal Krippendorff α was calculated post hoc as a supplementary between-reader agreement summary in the 115 treated image sets, separately for Experts 1–3 and the five AI configurations (Hayes and Krippendorff, 2007). Original three-category labels were retained without ordinal distance weighting. Alpha was 0.325 (95% CI 0.195 to 0.452) for experts and -0.099 (95% CI -0.131 to -0.065) for AI. Pointwise percentile intervals used the same 10,000 ordinary whole-participant resamples described above (seed 20260903), retaining all readings from each sampled participant together. In this complete, fixed-reader dataset, nominal α is the finite-sample counterpart of nominal Fleiss κ; their similarity is not independent validation. Alpha remains affected by pooled category frequencies and does not assess within-configuration repeatability or establish anatomical accuracy. The two groups were not compared with a hypothesis test.

S3.6 Descriptive error subgroups

Table S3.9A Errors by Sex

Stratum	Reader	n	Ref negative n	Three-class errors n (%)	Error % 95% CI
Female	Gemini 3.5 Flash	26	9	13 (50.0)	32.1–67.9
Female	Claude Sonnet 4.6	26	9	18 (69.2)	50.0–83.5
Female	GPT-5.5	26	9	17 (65.4)	46.2–80.6
Female	Grok 4.3	26	9	11 (42.3)	25.5–61.1
Female	Kimi K2.6	26	9	10 (38.5)	22.4–57.5
Female	Expert 1	26	9	1 (3.8)	0.7–18.9
Female	Expert 2	26	9	5 (19.2)	8.5–37.9
Female	Expert 3	26	9	3 (11.5)	4.0–29.0
Male	Gemini 3.5 Flash	89	20	39 (43.8)	34.0–54.2
Male	Claude Sonnet 4.6	89	20	48 (53.9)	43.6–63.9
Male	GPT-5.5	89	20	62 (69.7)	59.5–78.2
Male	Grok 4.3	89	20	23 (25.8)	17.9–35.8
Male	Kimi K2.6	89	20	45 (50.6)	40.4–60.7
Male	Expert 1	89	20	7 (7.9)	3.9–15.4
Male	Expert 2	89	20	15 (16.9)	10.5–26.0
Male	Expert 3	89	20	14 (15.7)	9.6–24.7

Table S3.9B Errors by BMI

Stratum	Reader	n	Ref negative n	Three-class errors n (%)	Error % 95% CI
<25 kg/m2	Gemini 3.5 Flash	22	3	10 (45.5)	26.9–65.3
<25 kg/m2	Claude Sonnet 4.6	22	3	16 (72.7)	51.8–86.8
<25 kg/m2	GPT-5.5	22	3	14 (63.6)	43.0–80.3
<25 kg/m2	Grok 4.3	22	3	4 (18.2)	7.3–38.5
<25 kg/m2	Kimi K2.6	22	3	12 (54.5)	34.7–73.1
<25 kg/m2	Expert 1	22	3	2 (9.1)	2.5–27.8
<25 kg/m2	Expert 2	22	3	2 (9.1)	2.5–27.8
<25 kg/m2	Expert 3	22	3	3 (13.6)	4.7–33.3
25 to <30 kg/m2	Gemini 3.5 Flash	72	22	34 (47.2)	36.1–58.6
25 to <30 kg/m2	Claude Sonnet 4.6	72	22	37 (51.4)	40.1–62.6
25 to <30 kg/m2	GPT-5.5	72	22	48 (66.7)	55.2–76.5
25 to <30 kg/m2	Grok 4.3	72	22	24 (33.3)	23.5–44.8
25 to <30 kg/m2	Kimi K2.6	72	22	34 (47.2)	36.1–58.6
25 to <30 kg/m2	Expert 1	72	22	6 (8.3)	3.9–17.0
25 to <30 kg/m2	Expert 2	72	22	13 (18.1)	10.9–28.5
25 to <30 kg/m2	Expert 3	72	22	12 (16.7)	9.8–26.9
>=30 kg/m2	Gemini 3.5 Flash	21	4	8 (38.1)	20.8–59.1
>=30 kg/m2	Claude Sonnet 4.6	21	4	13 (61.9)	40.9–79.2
>=30 kg/m2	GPT-5.5	21	4	17 (81.0)	60.0–92.3
>=30 kg/m2	Grok 4.3	21	4	6 (28.6)	13.8–50.0
>=30 kg/m2	Kimi K2.6	21	4	9 (42.9)	24.5–63.5
>=30 kg/m2	Expert 1	21	4	0 (0.0)	0.0–15.5
>=30 kg/m2	Expert 2	21	4	5 (23.8)	10.6–45.1
>=30 kg/m2	Expert 3	21	4	2 (9.5)	2.7–28.9

Sex strata contain 26 women and 89 men. BMI <25, 25 to <30 and ≥30 kg/m² strata contain 22, 72 and 21 participants, respectively, with only 3 and 4 reference-negative cases in the extreme BMI groups. Wilson intervals are descriptive; there were no subgroup tests or claims of fairness, equivalence or effect modification. BMI bands were selected post hoc.

S3.7 Missing data and observed clinical outcomes

Table S3.10 Source missingness and analysis coding in 115 treated participants

Variable	Original source blank n	Unavailable in analysis n	Coding note
Age	0	0	No imputation in the descriptive summaries; ventilation placeholders counted as unavailable when applicable.
Sex	0	0	No imputation in the descriptive summaries; ventilation placeholders counted as unavailable when applicable.
Weight	45	45	No imputation in the descriptive summaries; ventilation placeholders counted as unavailable when applicable.
Height	45	45	No imputation in the descriptive summaries; ventilation placeholders counted as unavailable when applicable.
Recorded BMI	0	0	No imputation in the descriptive summaries; ventilation placeholders counted as unavailable when applicable.
ASA physical status	0	0	No imputation in the descriptive summaries; ventilation placeholders counted as unavailable when applicable.
Hypertension	37	0	Presence-coded field; source blanks interpreted as absence, not individually verified negatives.
Diabetes mellitus	57	0	Present/absent coding confirmed by investigators; absent entries explicitly coded “-” in the revised working copy.
Asthma	113	0	Presence-coded field; source blanks interpreted as absence, not individually verified negatives.
Ejection fraction	0	0	No imputation in the descriptive summaries; ventilation placeholders counted as unavailable when applicable.
EuroSCORE II	0	0	No imputation in the descriptive summaries; ventilation placeholders counted as unavailable when applicable.
Alcohol use	112	0	Presence-coded field; source blanks interpreted as absence, not individually verified negatives.

Variable	Original source blank n	Unavailable in analysis n	Coding note
Smoking	60	0	Present/absent coding confirmed by investigators; absent entries explicitly coded “-” in the revised working copy.
Education	0	0	No imputation in the descriptive summaries; ventilation placeholders counted as unavailable when applicable.
HADS anxiety	0	0	No imputation in the descriptive summaries; ventilation placeholders counted as unavailable when applicable.
HADS depression	0	0	No imputation in the descriptive summaries; ventilation placeholders counted as unavailable when applicable.
Cross-clamp duration	0	0	No imputation in the descriptive summaries; ventilation placeholders counted as unavailable when applicable.
Intraoperative fentanyl	0	0	No imputation in the descriptive summaries; ventilation placeholders counted as unavailable when applicable.
Time to extubation	0	2	No imputation in the descriptive summaries; ventilation placeholders counted as unavailable when applicable.
Any postoperative complication	90	0	Presence-coded field; source blanks interpreted as absence, not individually verified negatives.
ICU length of stay	0	0	No imputation in the descriptive summaries; ventilation placeholders counted as unavailable when applicable.
Hospital length of stay	0	0	No imputation in the descriptive summaries; ventilation placeholders counted as unavailable when applicable.
Mortality	113	0	Presence-coded field; source blanks interpreted as absence, not individually verified negatives.
Resting NRS at 12 h	0	8	No imputation in the descriptive summaries; ventilation placeholders counted as unavailable when applicable.
Coughing NRS at 12 h	0	8	No imputation in the descriptive summaries; ventilation placeholders counted as unavailable when applicable.

Variable	Original source blank n	Unavailable in analysis n	Coding note
Resting NRS at 24 h	0	4	No imputation in the descriptive summaries; ventilation placeholders counted as unavailable when applicable.
Coughing NRS at 24 h	0	4	No imputation in the descriptive summaries; ventilation placeholders counted as unavailable when applicable.
Resting NRS at 48 h	0	2	No imputation in the descriptive summaries; ventilation placeholders counted as unavailable when applicable.
Coughing NRS at 48 h	0	2	No imputation in the descriptive summaries; ventilation placeholders counted as unavailable when applicable.
All nine reader classifications	0	0	Each reader had one recorded category for every treated participant.

Diabetes and smoking use the investigators' confirmed present/absent coding. In the other presence-coded fields, original blanks were interpreted as absence under the inherited convention, not individually verified negatives. Weight and height each have 45 source blanks, despite complete recorded BMI. Eight, four and two NRS values per outcome were unavailable at 12, 24 and 48 h. Two never-extubated participants had no observed NRS and died on ICU days 10 and 13. No self-report was imputed for them.

Table S3.11 Observed pain by reference group

Outcome and time	Successful, median (IQR); n	Patchy/inadequate, median (IQR); n
Rest, 12 h	2 (1–2); 79	2 (2–2.25); 28
Rest, 24 h	2 (1–2); 83	3 (2–3); 28
Rest, 48 h	2 (1–2); 85	3 (3–4); 28
Cough, 12 h	2 (2–3); 79	3 (3–4); 28
Cough, 24 h	3 (2–4); 83	4 (3.75–5); 28
Cough, 48 h	3 (2–4); 85	4.5 (4–5); 28

Table S3.12 Rescue receipt during the first 48 hours

Drug	Reference-successful n=86	Patchy/inadequate n=29
Paracetamol	27 (31.4%)	10 (34.5%)
Tramadol	16 (18.6%)	1 (3.4%)
Pethidine	2 (2.3%)	2 (6.9%)

These are participant receipt counts over 48 h, not total 24-h consumption or time-aligned exposure. Scheduled paracetamol was 1 g three times daily; rescue increments were 1 g paracetamol, 100 mg tramadol or 50 mg pethidine. Tramadol was more frequent in the reference-successful group. Unavailable administration timing prevents causal attribution or time-varying adjustment. Pain differences do not validate the anatomical reference.

S3.8 Exploratory ordinal models

Pain analyses were exploratory and used the nominal 12-, 24- and 48-hour assessment labels. Resting and coughing NRS were separately modelled with a cumulative-logit mixed model containing a normally distributed participant random intercept, flexible ordered thresholds, categorical time, reference-defined spread group, their interaction, sex, age, BMI, intraoperative fentanyl, cross-clamp time and HADS anxiety and depression. The model assumed proportional odds across the observed NRS cutpoints. For successful versus patchy/inadequate spread, we report association direction and exploratory pointwise inference rather than conditional odds-ratio magnitudes; these findings do not quantify NRS-point differences or clinical benefit. Maximum likelihood used all 331 observed values from 113 participants for each outcome under a conditional MAR assumption. The two never-extubated participants, both of whom died, had no self-report NRS and were excluded from pain modelling and imputation. Gaussian mixed-model results, including the expanded-confounder sensitivity, were retained as comparisons.

Maximum likelihood was implemented in Python using standard Gauss-Hermite quadrature with 161 nodes. Continuous covariates were standardised for numerical estimation. Validation included reproducing published cumulative-link mixed-model benchmark estimates and standard errors, independent adaptive integration of the study likelihood, numerical-score checks, multiple starting values, quadrature refinement and positive observed-information eigenvalues. This numerical validation does not establish the proportional-odds or missingness assumptions. Sparse extreme NRS levels limit formal assessment of proportional odds.

Table S3.13 Exploratory ordinal association direction and pointwise inference

Outcome	Nominal time	Association direction	Exploratory p
Rest	12 h	Lower NRS in reference-successful group	0.040
Rest	24 h	Lower NRS in reference-successful group	<0.001
Rest	48 h	Lower NRS in reference-successful group	<0.001
Cough	12 h	Lower NRS in reference-successful group	0.005
Cough	24 h	Lower NRS in reference-successful group	<0.001
Cough	48 h	Lower NRS in reference-successful group	<0.001

Inference is conditional on the specified covariates and participant random effect. Random-intercept standard deviations were approximately 4.6 and 4.7. Two-sided Wald p values are exploratory, pointwise and unadjusted for multiple comparisons. Direction and pointwise inference should not be interpreted as clinical effect magnitude. These post hoc models assume proportional odds and conditional MAR; numerical convergence does not validate these assumptions. No observed NRS was changed.

S3.9 Hypothetical informative-missingness sensitivity

A post hoc delta-adjusted ordinal pattern-mixture sensitivity was restricted to eight unavailable visits among the 113 participants with at least one observed NRS. All eight visits belonged to the reference-successful group; six were at the nominal 12-hour visit and two at 24 hours. It represented hypothetical self-report responses at those nominal visits, not measurements made during ventilation. The two participants without any observable NRS were never assigned pain values. Conditional probabilities from the observed-data ordinal mixed model were shifted by delta=-log(4), -log(2), 0, log(2), or log(4), corresponding to assumed multipliers of 0.25, 0.5, 1, 2 or 4 for the odds of higher pain among unavailable observations. Fifty datasets per scenario were generated with approximate posterior parameter draws and random-intercept draws conditional on observed responses, then refitted and combined using Rubin's rules on the log-odds scale. The same random draws across scenarios reduced Monte Carlo variation. These shifts were sensitivity assumptions, not estimated or clinically elicited differences. Imputation used only observed NRS categories; unobserved tail categories were not reconstructed. The analysis cannot recover actual assessment times or pain during ventilation and cannot establish that MAR holds.

Table S3.14 Delta-adjusted ordinal sensitivity scenarios

Outcome	Assumed missing-visit odds multiplier	Time	Association direction	Pointwise 95% interval excludes null
Rest	0.25	12 h	Lower NRS in reference-successful group	Yes
Rest	0.25	24 h	Lower NRS in reference-successful group	Yes
Rest	0.25	48 h	Lower NRS in reference-successful group	Yes
Rest	0.5	12 h	Lower NRS in reference-successful group	Yes
Rest	0.5	24 h	Lower NRS in reference-successful group	Yes
Rest	0.5	48 h	Lower NRS in reference-successful group	Yes
Rest	1.0	12 h	Lower NRS in reference-successful group	Yes
Rest	1.0	24 h	Lower NRS in reference-successful group	Yes
Rest	1.0	48 h	Lower NRS in reference-successful group	Yes
Rest	2.0	12 h	Lower NRS in reference-successful group	Yes
Rest	2.0	24 h	Lower NRS in reference-successful group	Yes
Rest	2.0	48 h	Lower NRS in reference-successful group	Yes
Rest	4.0	12 h	Lower NRS in reference-successful group	No
Rest	4.0	24 h	Lower NRS in reference-successful group	Yes
Rest	4.0	48 h	Lower NRS in reference-successful group	Yes
Cough	0.25	12 h	Lower NRS in reference-successful group	Yes
Cough	0.25	24 h	Lower NRS in reference-successful group	Yes
Cough	0.25	48 h	Lower NRS in reference-successful group	Yes
Cough	0.5	12 h	Lower NRS in reference-successful group	Yes
Cough	0.5	24 h	Lower NRS in reference-successful group	Yes
Cough	0.5	48 h	Lower NRS in reference-successful group	Yes
Cough	1.0	12 h	Lower NRS in reference-successful group	Yes
Cough	1.0	24 h	Lower NRS in reference-successful group	Yes
Cough	1.0	48 h	Lower NRS in reference-successful group	Yes
Cough	2.0	12 h	Lower NRS in reference-successful group	Yes
Cough	2.0	24 h	Lower NRS in reference-successful group	Yes
Cough	2.0	48 h	Lower NRS in reference-successful group	Yes
Cough	4.0	12 h	Lower NRS in reference-successful group	Yes
Cough	4.0	24 h	Lower NRS in reference-successful group	Yes
Cough	4.0	48 h	Lower NRS in reference-successful group	Yes

All 500 scenario-model fits converged. Direction and pointwise interval-based inference are reported without conditional odds-ratio magnitudes. The point estimates favoured lower NRS in the reference-successful group in

every displayed scenario. At 24 and 48 h, the pointwise 95% intervals excluded the null under all five selected shifts for both outcomes. At 12 h, the resting-NRS interval included the null under the fourfold-higher-pain scenario. These intervals are exploratory and unadjusted for multiple comparisons. This is sensitivity to selected hypothetical assumptions, not proof of robustness to all missing-not-at-random (MNAR) mechanisms or recovery of pain during ventilation. Imputation was confined to observed NRS-category support and excluded the two participants with no observable pain.

S3.10 Gaussian model comparisons

Generalised estimating-equation (GEE) sensitivity models used a Gaussian family with an identity link, the same core covariates and time-by-spread interaction as the mixed models, an exchangeable within-participant working correlation, and robust sandwich standard errors.

Table S3.15 Original Gaussian mixed-model contrasts

Outcome	Time	Adjusted difference, NRS points	95% CI
Rest	12 h	−0.353	−0.786 to 0.080
Rest	24 h	−0.793	−1.225 to −0.361
Rest	48 h	−1.208	−1.639 to −0.776
Cough	12 h	−0.687	−1.208 to −0.165
Cough	24 h	−1.160	−1.681 to −0.640
Cough	48 h	−1.301	−1.821 to −0.781

Table S3.16 Generalised estimating-equation (GEE) sensitivity contrasts

Outcome	Time	Difference, NRS points	Robust SE	95% CI
Rest	12h	-0.355	0.170	-0.688 to -0.021
Rest	24h	-0.793	0.190	-1.164 to -0.421
Rest	48h	-1.208	0.210	-1.619 to -0.796
Cough	12h	-0.687	0.204	-1.088 to -0.287
Cough	24h	-1.160	0.228	-1.608 to -0.712
Cough	48h	-1.301	0.207	-1.706 to -0.896

Table S3.17 Expanded-confounder Gaussian contrasts

Outcome	Time	Difference, NRS points	SE	95% CI
Rest	12h	-0.249	0.224	-0.687 to +0.190
Rest	24h	-0.689	0.223	-1.127 to -0.252
Rest	48h	-1.104	0.223	-1.541 to -0.666
Cough	12h	-0.467	0.264	-0.984 to +0.050
Cough	24h	-0.942	0.263	-1.458 to -0.426
Cough	48h	-1.083	0.263	-1.599 to -0.567

Table S3.17B Baseline characteristics by reference-defined spread group

Characteristic	Successful (n=86)	Patchy/inadequate (n=29)	Absolute standardised difference
Age, years	60.0 ± 9.0	59.8 ± 9.4	0.030
BMI, kg/m²	27.3 ± 3.2	27.5 ± 3.2	0.047
Ejection fraction, %	53.4 ± 9.3	55.2 ± 8.3	0.205
EuroSCORE II	1.4 ± 1.1	1.4 ± 0.7	0.043
HADS anxiety	7.1 ± 3.2	7.6 ± 4.1	0.147
HADS depression	5.4 ± 3.1	5.2 ± 3.8	0.061
Cross-clamp time, min	55.4 ± 17.2	58.8 ± 19.0	0.190
Intraoperative fentanyl, µg	536.6 ± 116.4	522.2 ± 133.7	0.115
Male sex	69 (80.2%)	20 (69.0%)	0.261
Hypertension	58 (67.4%)	20 (69.0%)	0.033
Diabetes mellitus	39 (45.3%)	18 (62.1%)	0.340
Asthma	0 (0.0%)	2 (6.9%)	0.385
Alcohol use	1 (1.2%)	2 (6.9%)	0.295
Smoking	44 (51.2%)	10 (34.5%)	0.342
Education: primary school	8 (9.3%)	2 (6.9%)	0.088
Education: secondary school	11 (12.8%)	9 (31.0%)	0.452
Education: high school	57 (66.3%)	18 (62.1%)	0.088
Education: university	10 (11.6%)	0 (0.0%)	0.513

The Gaussian models included the same core covariates as the ordinal models; the expanded model added diabetes, smoking, ejection fraction and categorical education. These are model comparisons rather than independent confirmatory analyses. Both 12 h intervals included zero with expanded adjustment. Figure S2 shows the Gaussian time-specific contrasts as secondary visualisation.

S3.11 Descriptive individual reader pain summaries

Table S3.18A Mean resting NRS by reader category

Rater	Successful n, median (Q1–Q3)	Not-successful n, median (Q1–Q3)
Gemini 3.5 Flash	76, 2.0 (1.3–2.7)	37, 2.0 (1.7–2.3)
Claude Sonnet 4.6	46, 2.0 (1.7–2.7)	67, 2.0 (1.3–2.8)
GPT-5.5	14, 1.8 (1.1–2.2)	99, 2.0 (1.3–2.7)
Grok 4.3	106, 2.0 (1.3–2.7)	7, 1.7 (0.8–2.2)
Kimi K2.6	33, 2.0 (1.3–3.0)	80, 2.0 (1.3–2.7)
Expert 1	83, 2.0 (1.2–2.3)	30, 2.7 (2.0–3.3)
Expert 2	85, 2.0 (1.0–2.3)	28, 2.5 (2.0–3.1)
Expert 3	84, 2.0 (1.2–2.7)	29, 2.3 (2.0–2.7)
Senior-expert final reference	85, 2.0 (1.0–2.3)	28, 2.7 (2.3–3.3)

Table S3.18B Mean coughing NRS by reader category

Rater	Successful n, median (Q1–Q3)	Not-successful n, median (Q1–Q3)
Gemini 3.5 Flash	76, 3.0 (2.6–4.0)	37, 3.0 (2.7–4.0)
Claude Sonnet 4.6	46, 3.3 (2.7–4.0)	67, 3.0 (2.7–4.0)
GPT-5.5	14, 3.0 (1.9–3.2)	99, 3.3 (2.7–4.0)
Grok 4.3	106, 3.0 (2.7–4.0)	7, 2.7 (1.8–3.8)
Kimi K2.6	33, 3.0 (2.7–4.0)	80, 3.0 (2.6–4.0)
Expert 1	83, 3.0 (2.0–3.5)	30, 4.0 (3.3–4.3)
Expert 2	85, 3.0 (2.0–3.7)	28, 3.8 (3.3–4.3)
Expert 3	84, 3.0 (2.2–4.0)	29, 3.7 (3.0–4.0)
Senior-expert final reference	85, 3.0 (2.0–3.3)	28, 4.0 (3.6–4.3)

Each participant's mean of the available nominal 12-, 24- and 48-h scores was calculated, then summarised across participants as median (Q1–Q3). These unadjusted summaries are descriptive; no correlation or Mann–Whitney significance tests are reported. Different readers generate different group sizes; these summaries do not establish comparative pain prediction.

S3.12 Direct comparisons with individual experts

Each of the five AI configurations was compared separately with each of Experts 1–3 in the 115 treated image sets. The comparator's category 1 is positive; both readers' categories 2 and 3 are combined as not successful. Table S3.19 provides the resulting 2 × 2 counts and raw binary agreement; the corresponding balanced agreement and pointwise intervals are in main Table 4. These calculations use the individual experts' labels, not the senior expert's final reference. The general individual-expert comparison was a secondary aim in the signed April protocol; the present treated-only binary implementation is exploratory and does not reproduce the protocol's three-category primary endpoint.

For each of the 15 pairs, pointwise percentile intervals use 10,000 participant resamples stratified by the selected expert's positive/negative class, retaining all readings for a sampled participant together. The fixed seed recipe is 20261012 + 100i + j, where AI indices i=0–4 follow the table order and expert indices j=0–2 denote Experts 1–3. Intervals condition on the recorded comparator and empirical category distribution; they do not include uncertainty in anatomical truth, guarantee simultaneous coverage or constitute new superiority tests. There are only 31, 29 and 30 expert-negative cases, respectively. For Grok versus Expert 3, no negative case matched (0/30); empirical resampling preserves this zero. Its percentile interval must therefore not be interpreted as a population inferiority test.

Table S3.19 Underlying direct AI–expert binary counts in 115 treated participants

AI configuration	Expert comparator	Expert positive/negative	TP/FN/FP/TN	Binary matches n (%)
Gemini 3.5 Flash	Expert 1	84/31	56/28/22/9	65/115 (56.5%)
Gemini 3.5 Flash	Expert 2	86/29	55/31/23/6	61/115 (53.0%)
Gemini 3.5 Flash	Expert 3	85/30	57/28/21/9	66/115 (57.4%)
Claude Sonnet 4.6	Expert 1	84/31	35/49/11/20	55/115 (47.8%)
Claude Sonnet 4.6	Expert 2	86/29	35/51/11/18	53/115 (46.1%)
Claude Sonnet 4.6	Expert 3	85/30	32/53/14/16	48/115 (41.7%)
GPT-5.5	Expert 1	84/31	12/72/2/29	41/115 (35.7%)
GPT-5.5	Expert 2	86/29	11/75/3/26	37/115 (32.2%)
GPT-5.5	Expert 3	85/30	11/74/3/27	38/115 (33.0%)
Grok 4.3	Expert 1	84/31	79/5/29/2	81/115 (70.4%)
Grok 4.3	Expert 2	86/29	80/6/28/1	81/115 (70.4%)
Grok 4.3	Expert 3	85/30	78/7/30/0	78/115 (67.8%)
Kimi K2.6	Expert 1	84/31	29/55/4/27	56/115 (48.7%)
Kimi K2.6	Expert 2	86/29	27/59/6/23	50/115 (43.5%)
Kimi K2.6	Expert 3	85/30	31/54/2/28	59/115 (51.3%)

TP/FN/FP/TN denote agreement/disagreement with the indicated expert labels, not known clinical truth. TP: both category 1; FN: expert category 1, AI category 2/3; FP: expert category 2/3, AI category 1; TN: both category 2/3. Counts retain each paired participant; no new observations or classifications were generated.

Table S3.19B Exploratory binary agreement between expert readers in 115 treated image sets

Expert pair first–second	Observed agreement % (95% CI)	Binary Cohen κ (95% CI)	Balanced agreement first reader / second comparator % (95% CI)	Balanced agreement second reader / first comparator % (95% CI)
Expert 1–Expert 2	75.7 (67.1–82.6)	0.369 (0.170–0.555)	68.9 (59.1–78.1)	68.1 (58.2–77.7)
Expert 1–Expert 3	78.3 (69.9–84.8)	0.442 (0.249–0.613)	72.4 (62.8–81.5)	71.9 (62.4–81.1)
Expert 2–Expert 3	67.8 (58.8–75.7)	0.157 (−0.038–0.344)	57.7 (48.1–67.7)	57.9 (48.1–67.7)

Successful corresponds to category 1; categories 2 and 3 were combined as non-successful. The five no-block controls were excluded. Observed-agreement intervals are Wilson 95% intervals. Unweighted binary Cohen κ intervals used 10,000 ordinary paired participant-level bootstrap resamples (seed 2026101303). Directional balanced agreement is the mean agreement within the comparator's successful and non-successful categories. Its intervals used 10,000 paired participant-level resamples stratified by that comparator's labels; with zero-based expert indices i and j, seeds were 2026101400 + 10i + j. Bootstrap intervals are pointwise percentile 95% intervals, not simultaneous intervals. Observed agreement and κ are symmetric; balanced agreement depends on which reader supplies the comparator labels. Neither expert is an anatomical gold standard. This exploratory benchmark is distinct from the three-category Fleiss κ and does not assess within-reader repeatability.

S3.13 Monthly study enrolment

Table S3.20 Monthly study enrolment by calendar month

Calendar month	Enrolled study participants
September 2025	5
October 2025	11
November 2025	9
December 2025	12
January 2026	15
February 2026	8
March 2026	11
April 2026	9
May 2026	40
**Total**	**120**

Counts describe the 120 enrolled study participants, including the five no-block image controls. The September 2025–April 2026 subtotal is 80; May 2026 contributes 40. These are study-enrolment counts, not hospital-wide surgical volumes, and the table does not supply individual enrolment dates.

S3.14 Method sources

Hayes, A.F.; Krippendorff, K. Answering the call for a standard reliability measure for coding data. Commun. Methods Meas. 2007, 1, 77–89. https://doi.org/10.1080/19312450709336664.

Christensen, R.H.B. A Tutorial on Fitting Cumulative Link Mixed Models with clmm2 from the ordinal Package. https://cran.r-project.org/web/packages/ordinal/vignettes/clmm2_tutorial.pdf (accessed 9 October 2026).

Tang, Y. Controlled pattern imputation for sensitivity analysis of longitudinal binary and ordinal outcomes with nonignorable dropout. Stat. Med. 2018, 37, 1467–1481. https://doi.org/10.1002/sim.7583. Correction: 2023, 42, 2027–2028. https://doi.org/10.1002/sim.9708. This source supports controlled-imputation principles; the present random-intercept approximate-posterior implementation is not Tang’s sequential/probit algorithm.

SAS Institute. Adjusting Imputed Values in Pattern-Mixture Models. https://support.sas.com/documentation/cdl/en/statug/68162/HTML/default/statug_mi_details63.htm (accessed 9 October 2026). The implemented shift applies equally across cumulative cutpoints.

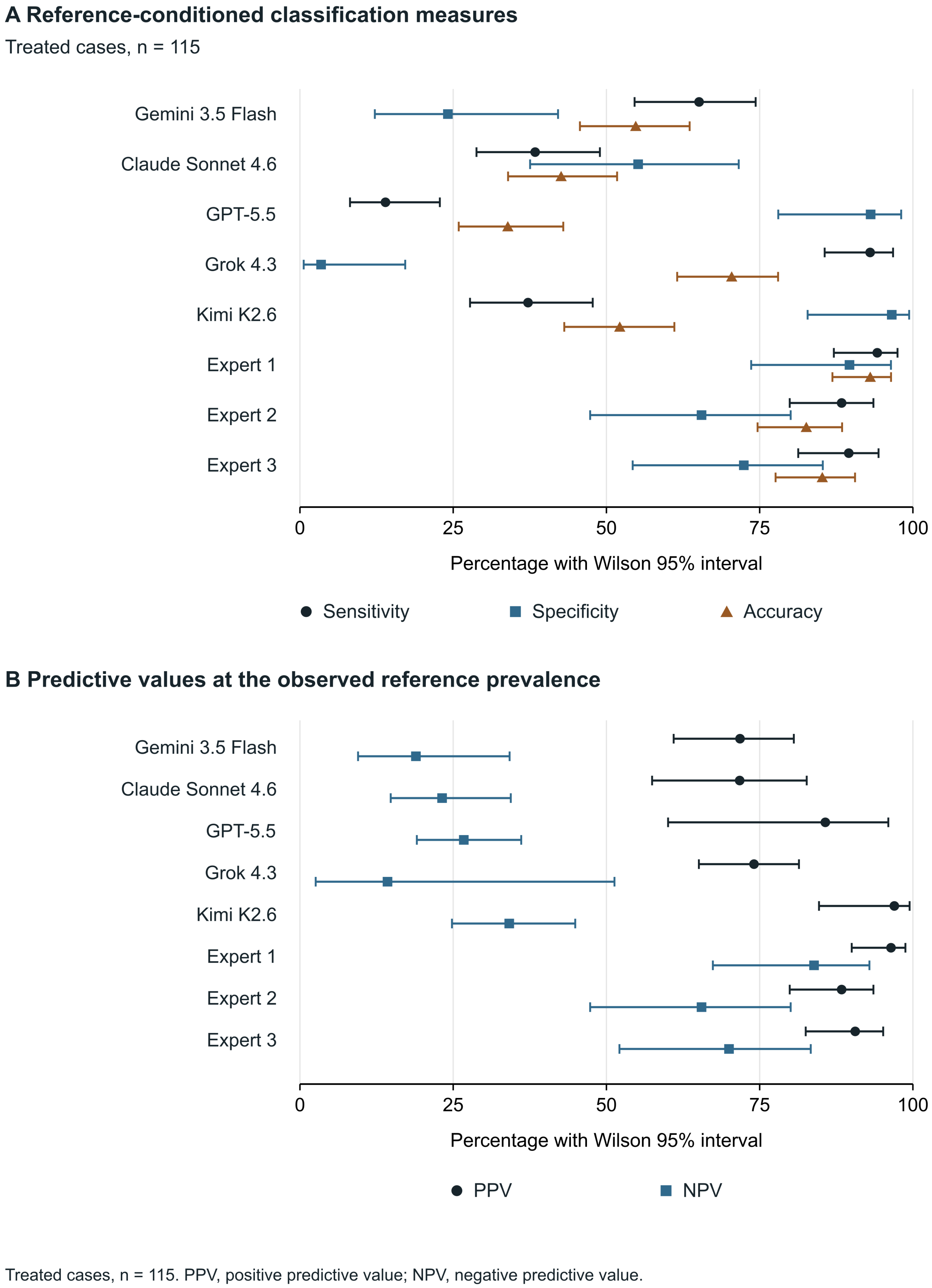

Figure S1 Secondary binary reference-conditioned measures in 115 treated participants. Wilson 95% intervals are shown. PPV and NPV depend on the reference-positive fraction 86/115.

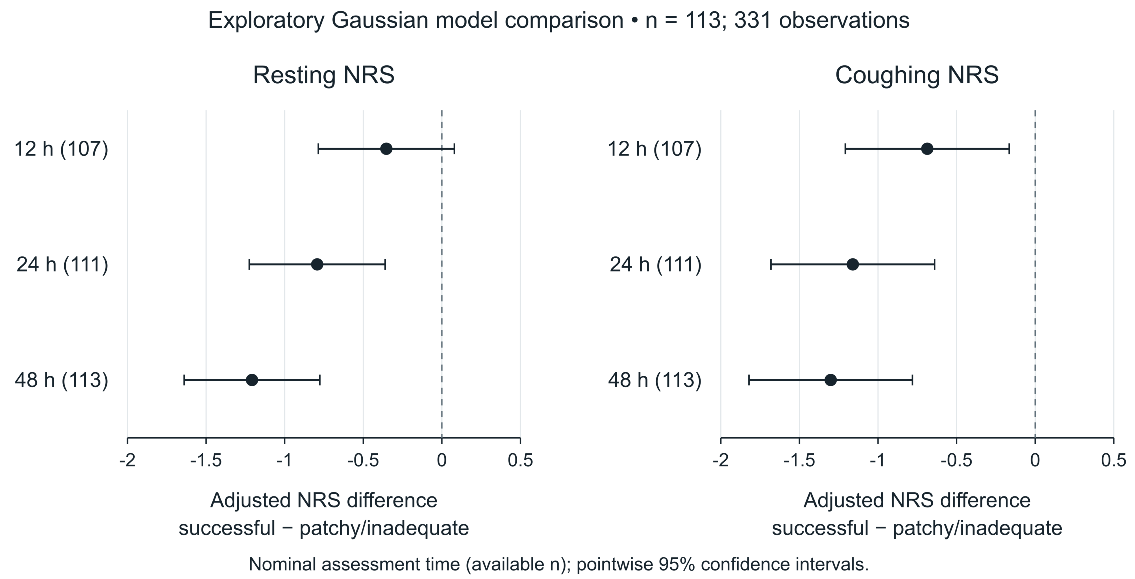

Figure S2 Exploratory Gaussian mixed-model contrasts for successful minus patchy/inadequate left-side spread. Negative differences denote lower NRS. Observations were available for 107 participants at 12 h, 111 at 24 h and 113 at 48 h; each outcome model uses 331 observations in 113 participants. Adjusted for sex, age, BMI, fentanyl, cross-clamp time and HADS anxiety/depression. This is not a causal analysis or reference validation.
