# Supplementary material for: Five Multimodal AI Configurations Versus Anaesthesiologists for Pecto-Intercostal Block Classification in an Archived-Image Reader Study of a Prospective CABG Cohort
Source: Diagnostics (Basel). 2026 Oct 28;16(21):3555. doi: 10.3390/diagnostics16213555 (PMC13762392; doi:10.3390/diagnostics16213555)
Supplement: Supplementary file 1 [file diagnostics-16-03555-s001.zip › 06_Supplementary_S4_Registry_Crosswalk.pdf]

Supplementary File S4 Protocol Registry and Manuscript Reconciliation

This reconciliation distinguishes the original research protocol dated 1 August 2025, the investigator-signed revised protocol and application dated 29 April 2026 (version 4), the earlier ClinicalTrials.gov record, the updated public record posted on 11 September 2026 and the manuscript submitted on that date. The clinical cohort was assembled prospectively, but registration and the September registry update were retrospective. The signed April protocol establishes what that revised document specified; it does not make the later treated-cohort binary endpoint identical to its three-category primary endpoint or establish prospective registration.

The initial Ethics Committee decision is dated 13 August 2025 and was transmitted on 14 August; its reviewed-document list identifies the protocol dated 1 August 2025, version 1. For the April revision, this reconciliation uses the investigator-signed protocol and application scans labelled version 4 and dated 29 April 2026. These replace the earlier editable April copies as the source for the revised protocol's content. The investigators identify the signed documents as the revised submission accompanying the LLM-method clarification and increase from 80 to 120 participants approved on 6 May 2026, before the June reader assessments. The protocol and application scans are distinguished from the committee's separate decision, which does not reproduce every methodological detail.

S4.1 Outcome hierarchy

Original protocol, signed April revision and earlier registry. The original protocol dated 1 August 2025 identifies the association between anatomical block score and postoperative NRS as primary, with reader agreement and analgesic consumption secondary. The signed revised protocol dated 29 April 2026 (version 4) changes this ordering: the primary aim is each of five LLMs' balanced accuracy for categories 1-3 against the senior adjudicator's final reference. Its statistical plan defines this measure as the mean of the three category-specific sensitivities. Pain associations, three-expert agreement and each LLM's comparisons with individual experts are secondary. The earlier public registry record retained postoperative NRS as primary and reader agreement as secondary, so it did not reflect the signed April protocol's objective hierarchy.

Updated record and manuscript. The update posted on 11 September lists binary balanced accuracy against the reference in 115 treated participants as primary and postoperative resting and coughing NRS as secondary. The September manuscript prioritised reader comparison. The current report treats the successful-versus-not-successful comparison in 115 treated participants as descriptive and analyses the five no-block controls separately. This binary measure is narrower than the three-category mean sensitivity specified in the signed April protocol. No treated participant had reference category 3, and the five no-block controls cannot supply observations of genuinely failed attempted injections. Therefore, the treated binary analysis and separate control findings are not presented as an unchanged execution of the protocol's primary endpoint. Three-category counts and agreement remain reported; combined all-120 descriptions are supplementary. Main Table 4 adds direct binary agreement between each AI configuration and each individual expert in the 115 treated participants. This addresses the general individual-expert secondary aim, but its narrower population, binary coding and intervals are exploratory. The later registry update does not establish prospective registration of these outcome definitions or of inferential analyses added during revision.

S4.2 AI method and reader design

Original protocol. The original protocol dated 1 August 2025 describes a trained CNN, including U-Net/ResNet approaches, training/validation/test partitions and comparison with a blinded anaesthesiologist. Our retained audit of the earlier registry identified a mixture of that trained-model plan and later descriptions of existing AI services.

Signed revised protocol dated 29 April 2026. Version 4 names the visual-input free consumer versions of Gemini, Claude, ChatGPT, Grok and Kimi, without model training or fine-tuning. It specifies the same fixed prompt, one query per model per case, and recorded category 1-3 outputs. Three experts read independently of one another and of the AI outputs. A senior adjudicator then considers their classifications when determining the final reference, while remaining blinded to AI outputs and clinical data. Thus, nine recorded classifications comprise five LLM outputs, three expert readings and one final adjudicated reference; they are not nine mutually independent

decisions. The investigators identify these documents as the revised submission accompanying the amendment approved on 6 May 2026. The decision letter mentions ChatGPT and Gemini in its rationale but does not reproduce the complete workflow.

Updated record and manuscript. The June 2026 reading exercise used five consumer multimodal AI configurations, three expert readers and a senior adjudicator's final reference. No bespoke AI model was developed or trained. The signed April protocol documents the five platform families and free consumer access, but does not independently verify the exact backend builds, session routing or query dates of the tested configurations. The protocol assigns categories 1, 2 and 3 to ideal, patchy and anatomically wrong blocks. It also explicitly provides for consented anatomical no-block recordings and says that their scores are image-based responses rather than evidence of an administered block. The report preserves this distinction and does not interpret the five controls as observed procedural failures. The revised report specifies that the senior expert reviewed the three experts' labels before giving the final reference decisions used in analysis.

The official record retained in the 9 October 2026 check uses the broader phrases 'assessed independently by all readers' and 'independently classified each image set'; these do not distinguish the expert-informed final reference analysed here. It also labels the ChatGPT configuration 'GPT-5.5 Instant in ChatGPT', whereas this report uses the investigators' confirmed label 'GPT-5.5'. These remaining reporting differences are disclosed here; the revised label is not independent verification of the June backend.

Score definitions. The original protocol assigned 1 to anatomically wrong and 3 to ideal blocks; the April protocol reversed those endpoints, retaining 2 for patchy blocks. The conducted classifications and analyses use 1 for successful, 2 for patchy/inadequate and 3 for failed/no local anaesthetic, as defined in the manuscript and Supplementary File S1.

Imaging and preparation. The original August protocol specifies a G-brand linear probe at 10-15 MHz and describes NYSORA-derived labelled examples and anatomical landmark preparation for model training. The signed April version 4 instead specifies a Mindray system with a 7.5 MHz linear probe, standardised depth, gain and focus, and use of existing LLMs without training or fine-tuning. Both documents list DICOM recording at a minimum resolution of 1920 x 1080; those planned acquisition specifications are not proof of the actual source-video format or dimensions of exported reader inputs. The conducted study used the Mindray M5/7L4s system at a nominal 7.5 MHz and supplied de-identified PNG frame exports to readers. No examples were supplied with the AI queries or as human-reader calibration material, and no formal prompt-development or pretesting dataset was documented. The signed April version does not contain the NYSORA preparation workflow described in the earlier editable April copy.

S4.3 Registration and study dates

Original and revised protocols and earlier registry. The original protocol gives a planned study period of 1 September 2025 to 1 April 2026. The revised protocol is dated 29 April 2026 and extends the planned end to 1 June 2026; the amendment decision is dated 6 May. The earlier public registry reported both primary and study completion as 1 May 2026.

Corrected record and manuscript. The record was first submitted on 6 May and first posted publicly on 12 May 2026, after recruitment began. The September correction was submitted on 9 September and posted publicly on 11 September. It records study start on 1 September 2025, primary completion on 27 May 2026 and study completion on 1 June 2026. The September manuscript already reported recruitment and imaging through 27 May, clinical follow-up and data collection by 1 June, and archived-image reading in June after follow-up. Registration remains retrospective relative to study commencement.

S4.4 Cohort and block procedure

Original protocol, signed April revision and earlier registry. Both protocols permit adults aged 18-80 years with ASA II-III status undergoing elective cardiac surgery through median sternotomy, including CABG, valve and combined procedures. The original August protocol and earlier registry describe SAPB plus PIFB or bilateral parasternal block after induction. The signed April version 4 specifies bilateral PIFB after sternal and skin closure. It also explicitly allows consented anatomical ultrasound recordings without a block when either the patient or surgical team declines block administration. The planned April bupivacaine volume is 30-40 mL of 0.25% solution in total.

Updated record and manuscript. The realised cohort comprised adults with ASA III status undergoing isolated CABG, a narrower population than the eligibility framework in either protocol. The 115 treated participants received bilateral PIFB after complete sternal and skin closure, using 20 mL of 0.25% bupivacaine per side (40 mL and 100 mg in total), as reported in the September manuscript. The procedure and timing agree with the signed April version, and the administered total volume is within its planned range. Five participants declined the block and supplied consented anatomical image sets; they are analysed as no-block controls rather than failed attempted blocks. These conducted-study descriptions also appear in the updated registry. The post-closure PIFB procedure was used throughout recruitment, including in treated participants enrolled before the 6 May 2026 amendment; it differed from the procedure described in the original August protocol.

S4.5 Pain assessments

Available protocol and earlier registry. The protocol copies describe NRS assessment at 0, 6, 12, 24 and 48 hours. The earlier public registry gave postoperative NRS primary status. The exact time-point text of the first submitted registry version was not independently retrieved for this reconciliation.

Corrected record and manuscript. The corrected registry lists separate resting and cough-evoked NRS outcomes at 6, 12, 24 and 48 hours, with the 6-hour assessment applicable only after extubation. The September manuscript analysed nominal 12-, 24- and 48-hour assessments and explained exclusion of the conditional 6-hour assessment. No 0-hour result was analysed. Actual assessment timestamps and predefined tolerance windows were unavailable; the reported hours are scheduled nominal time points.

S4.6 Rescue analgesia

Available protocol and earlier registry. Analgesic consumption was a secondary clinical aim in the available protocol. The earlier registry described a 24-hour analgesic-consumption outcome.

Corrected record and manuscript. The September manuscript already reported participant-level receipt of rescue paracetamol, tramadol and pethidine during the first 48 hours. The corrected registry describes these three drugs as separate receipt outcomes over the same interval. The available reporting records and unreliable administration timestamps do not permit reconstruction of a reliable 24-hour total-consumption outcome. The 48-hour receipt results therefore do not fulfil the earlier 24-hour consumption measure.

S4.7 Time to first mobilisation

Available protocol and earlier registry. Time to first mobilisation appeared among the planned clinical variables and in the earlier registry secondary outcomes.

Corrected record and manuscript. We did not collect this variable, so no result is available. It is absent from the corrected registry outcome set and is disclosed here as an uncollected planned outcome. Removal from the registry does not supply the missing outcome data.

S4.8 Enrolment amendment and recruitment intensity

Available documents and confirmed sequence. The original protocol and application target 80 participants; the signed April version 4 targets 120. The signed 6 May decision explicitly approves the increase from 80 to 120. The investigators confirm that the LLM-method clarification belonged to the same amendment and that participants 81-120 were enrolled after approval, as stated in the September manuscript. The final surgery and image acquisition occurred on 27 May. The updated registry reports 120 participants, comprising 115 treated participants and five no-block image controls. The original protocol cites a pilot-based calculation, while the signed April protocol asserts that n=120 increases power above 90%; the supplied documents do not contain enough assumptions to reconstruct either claim as a power justification for the current 115-participant binary reader comparison.

Recruitment explanation and monthly enrolment. Faster study recruitment reflected changes in the anaesthesia team's work organisation and greater availability for study-related block performance and ultrasound recording, not an increase in routine surgical volume. The same consultant anaesthesiologist continued to perform the study blocks and acquire the recordings, and recruitment remained restricted to operator-available days. Supplementary Table S3.20 reports monthly study enrolment: 80 participants from September 2025 through April 2026 and 40 in May 2026, for a total of 120.

The current official ClinicalTrials.gov record for NCT07581678 was checked on 9 October 2026. It identifies 9 September 2026 as the last update submission date and 11 September 2026 as the public posting date.

Descriptions of the earlier registry follow the retained audit performed on 3 September 2026 of Version 2, submitted on 16 July and posted on 17 July 2026. The exact contents of the first submitted version were not independently retrieved for this reconciliation.

The manuscript comparison uses the version submitted on 11 September 2026. The original protocol dated 1 August 2025 and its application are available as electronic copies. The revised protocol and application are now represented by the author-provided investigator-signed scans labelled version 4 and dated 29 April 2026. These scans, not the earlier editable April copies or their inconsistent internal version fields, are the source for the revised-protocol descriptions in this reconciliation. The source documents are preserved without alteration.

The confidential supporting set also includes the 13 August 2025 decision, its 14 August transmittal letter and the 6 May 2026 decision. The 6 May original and its unofficial English translation had already been supplied to the editor with the September resubmission. Translations are distinguished from the Turkish originals and do not enlarge their scope. Investigator signatures on the revised protocol and application are distinguished from the committee's decision; no independent authentication of signatures or committee archive verification is claimed.

AI, artificial intelligence; ASA, American Society of Anesthesiologists physical status; CABG, coronary artery bypass grafting; CNN, convolutional neural network; LLM, large language model; NRS, numerical rating scale; PIFB, pecto-intercostal fascial plane block; SAPB, serratus anterior plane block.

ClinicalTrials.gov study record NCT07581678: https://clinicaltrials.gov/study/NCT07581678

ClinicalTrials.gov structured study record checked 9 October 2026: https://clinicaltrials.gov/api/v2/studies/NCT07581678

S4.9 Summary comparison

Table S4.1 Summary of protocol registry and report differences

Topic	Original August 2025 protocol and earlier registry	Signed April 2026 protocol version 4	Updated registry posted 11 September 2026	Current report
Outcome hierarchy	August protocol: block-score–NRS association primary; reader agreement and analgesic consumption secondary. Earlier registry retained pain as primary.	Each LLM's three-category balanced accuracy against the final adjudicated reference primary; pain, expert agreement and LLM–expert comparisons secondary.	Binary balanced accuracy in 115 treated participants primary; resting and coughing NRS secondary.	Principal descriptive final-reference binary comparison in 115 treated participants; five no-block controls separate. Direct AI–individual-expert binary agreement added as an exploratory secondary analysis. Neither binary analysis is presented as unchanged execution of the April three-category primary endpoint.
AI and reference design	August: trained CNN with training/validation/test partitions. Earlier registry mixed this plan with descriptions of existing AI services.	Five free consumer LLM platforms, no training, single fixed-prompt query per case. Senior expert determines the final reference after reviewing three expert labels.	Reader comparison using existing AI services; no bespoke model development. The retained 9 October record uses broader independence wording and the label GPT-5.5 Instant in ChatGPT.	June consumer-configuration snapshot; final reference informed by expert labels and therefore non-independent. Exact backend builds and raw AI responses were not retained.
Sample and eligibility	Planned n=80; adults aged 18–80 years, ASA II–III, elective cardiac surgery through median sternotomy.	Planned n=120; broad cardiac-surgery eligibility retained.	Enrolment 120; conducted cohort described.	120 ASA III isolated-CABG participants: 115 received PIFB and five declined the block. Available-day consecutive recruitment; broader unscreened population unquantified.
Block procedure	August protocol and earlier registry: SAPB plus PIFB/bilateral parasternal block after induction.	Bilateral PIFB after sternal and skin closure; total 30–40 mL of 0.25% bupivacaine; consented anatomical no-block recordings allowed.	PIFB after surgical closure; actual procedure described.	Bilateral PIFB after complete sternal and skin closure: 20 mL of 0.25% bupivacaine per side, total 40 mL/100 mg. Controls are not failed attempted blocks.
Dates	August planned period: 1 September 2025–1 April 2026. Earlier registry: primary and study completion 1 May 2026.	Planned end extended to 1 June 2026; signed 29 April; amendment decision 6 May.	Start 1 September 2025; primary completion 27 May 2026; study completion 1 June 2026.	Recruitment/imaging through 27 May; clinical follow-up/data collection by 1 June; archived-image reading in June. Registration remains retrospective.

Topic	Original August 2025 protocol and earlier registry	Signed April 2026 protocol version 4	Updated registry posted 11 September 2026	Current report
Pain schedule	Protocols specified 0, 6, 12, 24 and 48 h. Earlier registry gave pain primary status; earliest-version time-point text was not independently retrieved.	The planned 0-, 6-, 12-, 24- and 48-h schedule remained.	Resting and coughing NRS at 6, 12, 24 and 48 h; 6 h conditional on extubation.	Nominal 12-, 24- and 48-h NRS analysed. Conditional 6-h assessment excluded; no 0-h result analysed. Actual assessment timestamps unavailable.
Rescue analgesia	Analgesic consumption secondary; earlier registry specified 24-h totals.	Analgesic consumption remained secondary.	Receipt of rescue analgesia within the first 48 h.	Drug-specific receipt within 48 h, not cumulative 24-h dose. Timing data do not permit reconstruction of the older 24-h endpoint.
Mobilisation	Planned clinical variable and earlier registry outcome.	Planned clinical follow-up variable.	Removed from the corrected outcome list.	Not collected; reported as an uncollected planned outcome, not silently analysed or substituted.
